# A memory switch for plant synthetic biology based on the phage ϕC31 integration system

**DOI:** 10.1093/nar/gkaa104

**Published:** 2020-02-21

**Authors:** Joan Miquel Bernabé-Orts, Alfredo Quijano-Rubio, Marta Vazquez-Vilar, Javier Mancheño-Bonillo, Victor Moles-Casas, Sara Selma, Silvia Gianoglio, Antonio Granell, Diego Orzaez

**Affiliations:** Instituto de Biología Molecular y Celular de Plantas (IBMCP). CSIC - Universidad Politécnica de Valencia. Camino de Vera s/n, 46022 Valencia, Spain

## Abstract

Synthetic biology has advanced from the setup of basic genetic devices to the design of increasingly complex gene circuits to provide organisms with new functions. While many bacterial, fungal and mammalian unicellular chassis have been extensively engineered, this progress has been delayed in plants due to the lack of reliable DNA parts and devices that enable precise control over these new synthetic functions. In particular, memory switches based on DNA site-specific recombination have been the tool of choice to build long-term and stable synthetic memory in other organisms, because they enable a shift between two alternative states registering the information at the DNA level. Here we report a memory switch for whole plants based on the bacteriophage ϕC31 site-specific integrase. The switch was built as a modular device made of standard DNA parts, designed to control the transcriptional state (on or off) of two genes of interest by alternative inversion of a central DNA regulatory element. The state of the switch can be externally operated by action of the ϕC31 integrase (Int), and its recombination directionality factor (RDF). The kinetics, memory, and reversibility of the switch were extensively characterized in *Nicotiana benthamiana* plants.

## INTRODUCTION

Plant synthetic biology is an established but continuously growing field that combines the engineering principles of decoupling, abstraction, and standardization with plant biology to provide plant systems with new traits and functions (1). Plants naturally offer a myriad of metabolic resources that can be harnessed by modern synthetic biology to tackle current challenges in the food supply chain, energy production, pest management, or manufacturing of valuable biologicals for medicine and industry (2). Some recent efforts in plant synthetic biology have introduced new metabolic pathways and gene circuits to improve relevant traits such as crop productivity, vitamin fortification (3,4), fixation of atmospheric nitrogen (5), or photosynthesis (5–7). While significant advances are being made, introducing novel or improved functions into plant systems often results in unpredicted adverse effects due to the complexity of their metabolism and signaling networks (8). Engineering new functions into living organisms requires precise control over the synthetic gene circuits to ensure optimal use of the metabolic resources available, while minimizing collateral deleterious effects. Synthetic biologists have developed a variety of regulatory tools to strictly control complex circuits, which has enabled the development of finely-tuned designed functions in bacterial and mammalian systems (9). This success stemmed from the extensive characterization of standard genetic devices and molecular tools that can be combined to build increasingly complex functions. The implementation of such complex circuits in plants has been more challenging due to the limited availability of well-documented genetic elements as well as the technical limitations of high-throughput plant transformation and circuit characterization (1,8,10). Therefore, current efforts in plant synthetic biology are aimed to develop reliable DNA assembly standards (phytobricks) to allow more efficient biodesign of genetic elements that can be used to build highly regulated synthetic plant functions (11,12).

Early synthetic regulatory devices relied on the transient use of activators and repressors to control transcription, which required a constant supply of inducers to sustain the desired cell output (13). Several chemically-inducible systems have been adapted to plant biotechnology to achieve transcriptional control, such as ethanol (14), glucocorticoids (15,16), copper (17,18), or insecticide-inducible promoters (19). However, these rely on the continuous addition and monitoring of external chemical inducers, which can be expensive, harmful to the environment, and challenging to control in large crop fields. In contrast, synthetic memory devices provide the ability to respond to limited external inputs with a sustained response. Memory devices have been pivotal for the design of complex genetic circuits in synthetic biology (9,20,21), but memory devices adapted and/or engineered for the plant chassis are almost absent. As a notable exception, Müller *et al.* (22) developed a transcriptional toggle switch for plant cell protoplasts, based on a transcriptional activator regulated by red light. This device is based on the slow dissociation rate of a red light-induced protein-protein interaction, which provides precise spatiotemporal control of gene expression but makes it susceptible to protein decay and dilution (i.e. transient memory) (20). The development of new memory devices for plants with long-term and heritable memory storage will open a door for new plant synthetic biology applications, by enabling precise control over agronomically-relevant outputs such as flowering time, stress response, or the biosynthesis of added value metabolites.

**Figure 1.**
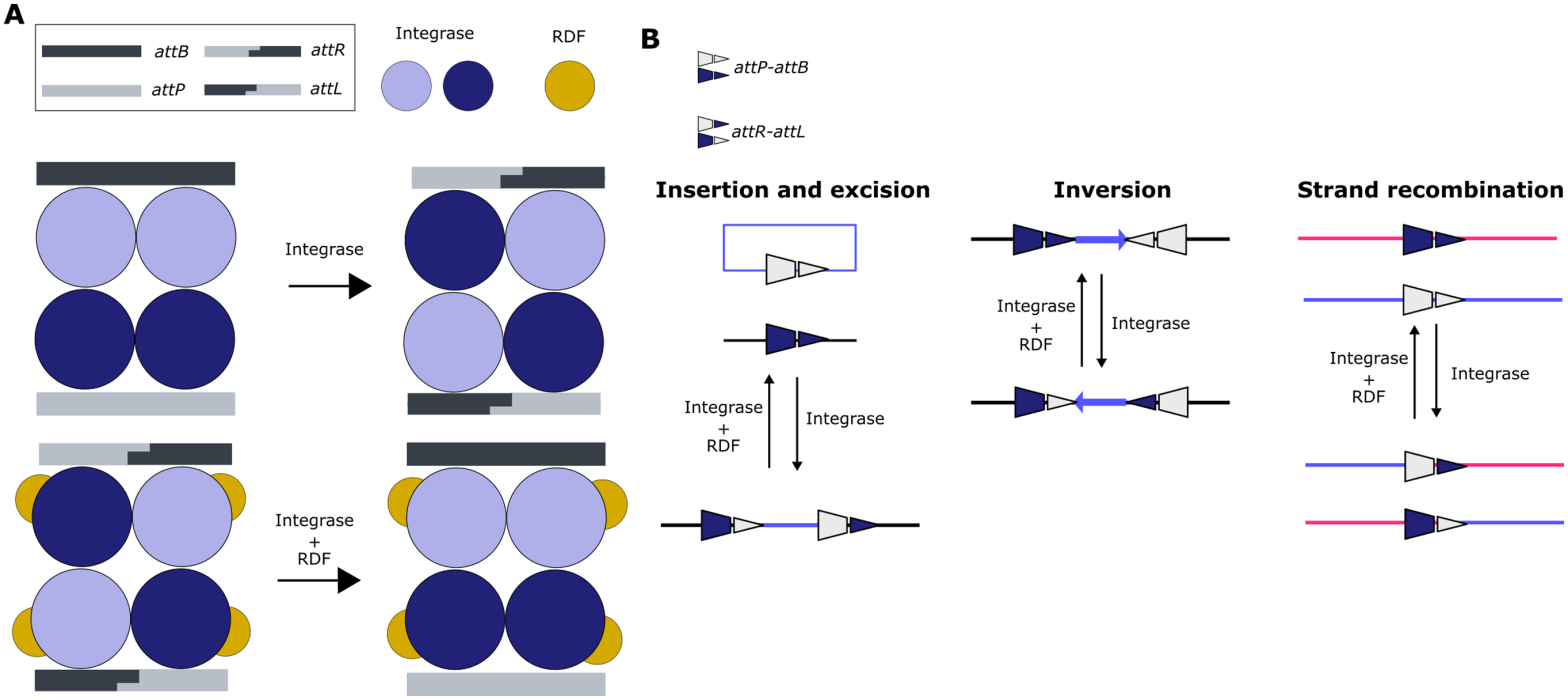
Mechanism of site-specific DNA recombination by Serine integrases. (**A**) Upper panel: In solution, serine integrases form dimers with affinity to the specific DNA attachment sites *attB* and *attP*. Binding to the *att* sites induces a conformational change that causes the tetramerization of the binary DNA-Integrases complexes. This triggers the cleavage of both DNA strands leaving 2-bp complementary overhangs. Next, the integrase subunits rotate 180° relative to each other, resulting in the strand exchange of the split *att* sites. Finally, the half-sites are re-ligated creating hybrid *attR* and *attL* sites. Lower panel: This reaction can be reversed in the presence of the recombination directionality factor (RDF), an allosteric modulator that binds to the integrase allowing the reaction to take place in the opposite direction. (**B**) Possible outcomes of the site-specific recombination process depending on the orientation of the *att* sites and the topology of the DNA molecules involved. In this work, the inversion mechanism between oppositely-oriented *att* sites on the same DNA strand has been exploited to engineer a reversible memory switch for plant synthetic biology.

Synthetic gene memory can be built through diverse mechanisms, such as transcription-based double-negative feedback loops and positive feedback loops, but DNA site-specific recombination has been the tool of choice to engineer long-term heritable memory (23). Site-specific recombination is mediated by Integrases, a group of enzymes found in temperate bacteriophages that catalyze the recombination of *attP* and *attB* DNA attachment sites, generating hybrid *attR* and *attL* sites in a strictly unidirectional reaction (24). This process can be reversed in the presence of the recombination directionality factor (RDF), an allosteric modulator of the integrase activity, which enables the recombination of the *attR-attL* sites back to *attP-attB* again (25,26) (see Figure 1A). This specific DNA recombination event can result in a variety of DNA-rearrangements (integration, excision, inversion, and translocation) depending on the topology of the initial DNA molecules and the orientation of the recombination sites (Figure 1B). Site-specific recombination mechanisms of diverse integrases have been extensively exploited in bacteria and mammalian cells to engineer reversible synthetic memory devices and complex logic circuits. Some examples of their application include counting cellular events (27,28), storing and rewriting biological data in the chromosome (29,30), creating state machines that implement two-input Boolean logic functions (31,32), and engineering complex regulatory circuits (32–34). On the other hand, the use of site-specific recombination tools in plants has been limited to transgene engineering for crop breeding applications such as removal of foreign DNA (35,36), stacking agronomic-valuable traits (37,38), and chloroplasts engineering (35); but their use for building synthetic memory devices in plants remains unrealized (39,40).

**Figure 2.**
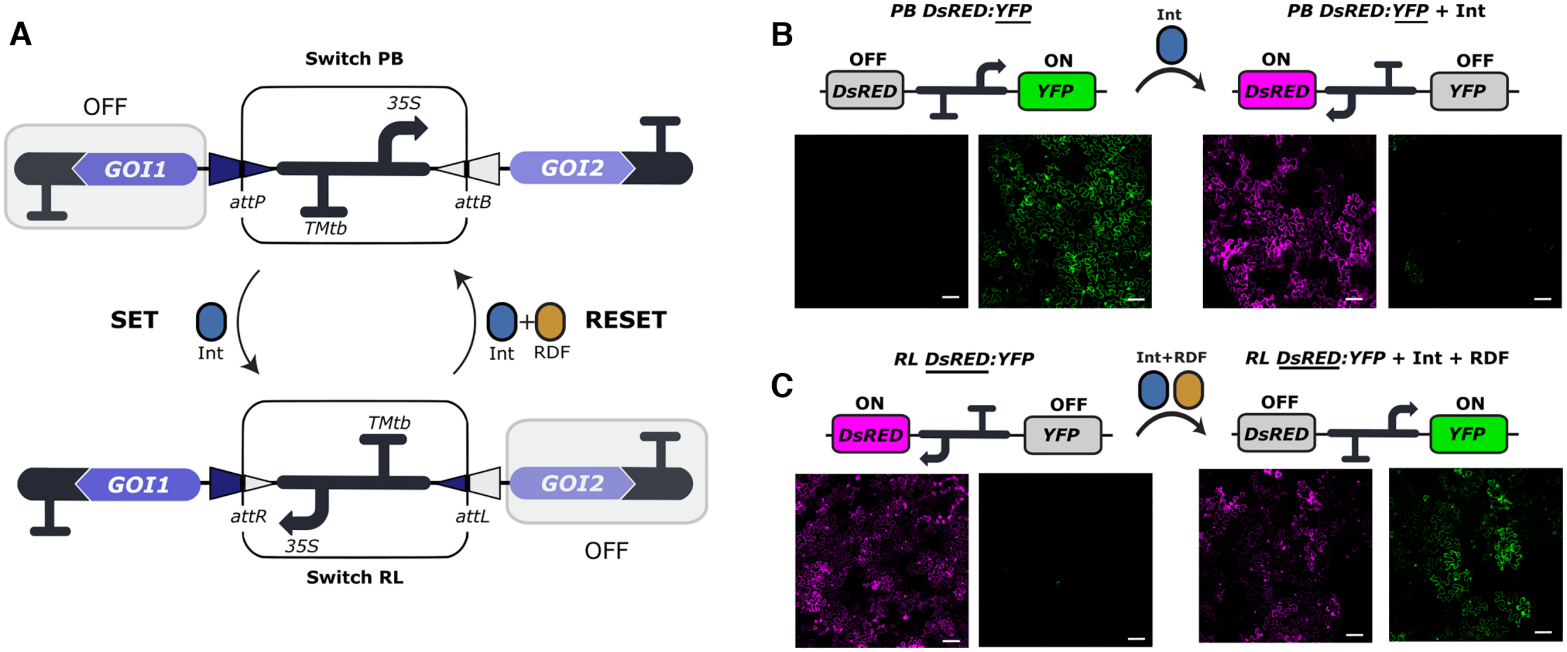
Design and functional validation of the plant switch based on the bacteriophage ϕC31 integration system. (**A**) An invertible plant promoter element works as a switch for the regulated expression of two genes of interest (*GOI1* and *GOI2*). The promoter orientation can be inverted by the action of the ϕC31 integrase (Int), which catalyzes site-specific recombination of the *attP* and *attB* sites flanking the promoter (SET operation). This event results in a change in the expression status of the two *GOIs* and the creation of the chimeric *attR* and *attL* sites. Expression of the Int and recombination directionality factor (RDF) catalyzes the recombination of *attR* and *attL* to reset the switch to its original state (RESET operation). The genetic parts encoding the PB state of the switch (GB1494), the RL state of the switch (GB1506), the Int (GB1531) and the RDF (GB1508) can be found in the GoldenBraid collection, using the identifiers between brackets. (**B, C**) Confocal laser microscopy images of WT *N. benthamiana* leaves infiltrated with (B) the *PB DsRED:YFP* (GB1495) or (C) the *RL DsRED:YFP* (GB1510) switch alone (left half of the panel) or the same switches in combination with (B) Int or (C) Int + RDF (right half of the panel). In each pair of images the left shows DsRED fluorescence and the right shows YFP. Images represent a tile of nine individual pictures that were taken 3 days post infiltration. Fluorescent proteins are spread throughout the nuclei and cytoplasm of the cells. The scale bar represents 100 μm.

Here, we present the first reversible memory switch for whole plants based on the bacteriophage ϕC31 serine integrase (herein referred to as Int) and its cognate recombination directionality factor, RDF (26). The switch is designed to control the transcription of two genes of interest (Figure 2). In the initial state of the switch (PB configuration), the gene located at the right side of the device is transcriptionally active (ON), while the second one (left) remains inactive (OFF). A limited supply of the Int catalyzes the inversion of the central DNA regulatory element, thus, changing the state of the switch from PB to RL configuration (this reaction is defined as SET operation). The new RL status is maintained until intentionally reversed to PB state (RESET operation) by a combined supply of Int and RDF. Hereinafter, we refer to these DNA constructs as *PB GOI1:GOI2* and *RL GOI1:GOI2*, where the state of the switch is denoted by the letters PB or RL, and the active gene of interest (*GOI1* or *GOI2*) is underlined. The components of the genetic switch were adapted for expression in the plant chassis and standardized using the GoldenBraid DNA assembly platform to allow easy repurposing of this tool to other plant synthetic biology applications (11,26). We analyzed the kinetics, memory, and reversibility of this genetic device in whole *N. benthamiana* plants through transient and stable transformation experiments using transgenic plants and hairy roots. Additionally, we coupled the integrase expression to an estradiol-inducible promoter as a proof of principle of externally-induced activation of the switch.

## MATERIALS AND METHODS

### Cloning and assembly of the GoldenBraid constructs

All the constructs used in this work were assembled using the GoldenBraid assembly platform (11). Briefly, in the GoldenBraid cloning schema, Level 0 basic DNA parts such as the Int, RDF, PB and RL switches were PCR-amplified or synthesized and then cloned into the pUPD2 plasmid through a BsmBI-mediated restriction-ligation reaction. We refer to this process as domestication. These cloned basic DNA parts were subjected to restriction reaction and Sanger sequencing for confirmation. Confirmed DNA parts were then used to assemble transcriptional units in Level 1 through a BsaI-mediated restriction-ligation reaction using pDGB3 α as destination vector. These transcriptional units were combined to create increasingly complex genetic modules in Level >1, by using BsaI- and BsmBI-mediated assembly reactions into two destination vectors (pDGB3 α and pDGB3 Ω). Transcriptional units and genetic modules were confirmed by restriction reactions. Detailed protocols for these procedures can be found elsewhere (41,42). The assembly of the switches described in this study required non-conventional GoldenBraid domestication of the DNA elements to be used as genes of interest (*GOI1* and *GOI2*) (see ‘Supplementary Materials and Methods’ section). Subsequently, the switch constructs were assembled by setting up a tripartite BsaI restriction-ligation reaction using domesticated *GOI1* + PB or RL switch + *GOI2*. A complete list of all constructs used in this work can be found in Supplementary Table S1, and their sequence information and assembly history are available at the GoldenBraid online repository (https://gbcloning.upv.es/). The list of oligonucleotides used to build them can be found in Supplementary Table S2.

### Agroinfiltration experiments

Transient expression experiments were performed to test the SET and RESET recombination operations as described in the main text. Equal volumes of *Agrobacterium* cultures carrying the different versions of the switches (*PB LUC:YFP, PB YFP:LUC, RL LUC:YFP* and *RL YFP:LUC*) and their appropriate operators, Int (SET), Int + RDF (RESET) or Stuffer Fragment as a negative control (SF, an empty vector containing a tomato intragenic region), were mixed and agroinfiltrated in wild type (WT) *N. benthamiana* plants. For the transgenic lines, only the aforementioned operators or P19 silencing suppressor as negative control were used. These cultures were first grown from glycerol stocks for two days until saturation, then 10 μl were sub-cultivated in 5 ml of LB media with the corresponding antibiotic and grown for 16h. Next, the cultures were pelleted, resuspended in the agroinfiltration buffer (10 mM MES, pH 5.6, 10 mM MgCl_2_ and 200 μM acetosyringone) and adjusted to the appropriate optical density (see Supplementary Table S3 for details). Four to five weeks-old *N. benthamiana* plants cultivated under a 24°C (light)/20°C (darkness) 16-h-light/8-h-dark photoperiod were used.

For reporter gene expression analysis, one plant per experimental point was used (from 1 to 7 days post infiltration (dpi)). Three leaves per plant were agroinfiltrated. Each leaf was infiltrated in two different spots, one spot with the recombination mixture and the other spot with the negative control. At each time-point, the corresponding plant was sampled. The agroinfiltrated spots of each leaf were sampled using a 0.8 cm cork-borer. For the quantitative assays, one disc (∼20 mg of tissue) was collected for each agroinfiltrated spot, giving six samples per plant (three switched plus three controls). All the discs were placed in 2 ml Eppendorf tubes and immediately frozen in liquid nitrogen. Samples were ground with a Retsch Mixer Mill MM400 for 1 min at 30 Hz and stored at −80°C for subsequent analysis.

### Quantification of reporter gene expression

Firefly (Fluc) and Renilla (Rluc) luciferase activities were determined using the DualGlo^®^ Luciferase Assay System (Promega) manufacturer's protocol with minor modifications. Homogenized leaf disc samples were extracted with 375 μl of ‘Passive Lysis Buffer’, vortexed, and then centrifuged for 10 min (14 000 × g) at 4°C. Then, 7.5 μl of supernatant were transferred to a 96-well plate and 30 μl of LARII added to quantify the Fluc activity. Finally, 30 μl of Stop & Glo Reagent was used to measure the Rluc activity. This latter inhibits the Fluc activity and contains the substrate for the subsequent Rluc-catalyzed reaction. Measurements were made using a GloMax 96 Microplate Luminometer (Promega) with a 2 s delay and a 10 s measurement. Fluc/Rluc ratios were determined as the mean value of three biological replicates, coming from three independent agroinfiltrated leaves of the same plant.

YFP quantifications in transgenic *N. benthamiana* plants were performed in a VICTOR X5 2030 fluorimeter (PerkinElmer), using F485 excitation filter and F535-40 emission filter. Individual samples made of three leaf discs were ground in 150 μl of PBS buffer, vortexed, and centrifuged at 13 000 rpm for 10 min at 4°C. Lastly, 10 μl of the extract were transferred to 96-well plates and measured with the spectrofluorometer. The YFP values are expressed as the mean of the measure of three independent leaves from the same plant.

### Confocal laser microscopy

Leaves of *N. benthamiana* plants agroinfiltrated with *RL DsRED:YFP* or *PB DsRed:YFP* were examined 5 days post infiltration under a ZEISS 780 AxioObserver Z1 confocal laser microscope equipped with C Apo 40×/1.2 W lens (water immersion) to visualize the yellow fluorescent protein YFP (λ_ex_ = 514 nm; λ_em_ = 518–562 nm) and DsRED (λ_ex_ = 561 nm; λ_em_ = 563–642 nm) fluorescence. Images of 9–16 tiles (one μm depth of focus each) were taken to visualize a larger area and processed with the ZEN 2.5 lite and FIJI software. Brightness and contrast of the unswitched negative controls (P19) and their corresponding switched samples (Int, Int + RDF-treated or Int-RDF) were equally adjusted to ensure their comparability. Adjustments made for different switches and/or transitions were not necessarily the same; therefore, comparisons of fluorescence intensity are only valid for each transition.

### Genotyping and quantitative PCR analysis

For all PCR analyses, genomic DNA (gDNA) was extracted following the CTAB protocol (43). For the qualitative diagnosis of the switch status, gDNAs were PCR-amplified with MyTaq™ DNA Polymerase (Bioline) using specific primers for the integrase (Int) and PB or RL configurations (Figure 4B and Supplementary Table S2). DNA amplification was confirmed on 1% agarose gel electrophoresis. For quantification of SET and RESET reactions at the DNA level, quantitative PCR analysis (qPCRs) were performed. qPCR samples were collected from three consecutive plant leaves infiltrated with the recombination mixture (Int, Int + RDF or Int-RDF). Two additional leaves were separately infiltrated with an *Agrobacterium* culture containing only P19 silencing suppressor and used as negative controls. One leaf disc per agroinfiltrated leaf (approx. 20 mg of tissue) was collected for each time-point (from 1 to 6 dpi) and extracted as described above. gDNA samples were then analyzed by qPCR in the Applied Biosystems 7500 Fast Real Time PCR System. Reactions were performed using a SYBR^®^ Premix Ex Taq (Takara) following the manufacturer's protocol. PCRs were run in triplicates using 30 ng of gDNA as template. A specific set of primers was used to amplify the RL state (MV1F1 and MV2R1, Supplementary Table S2). An additional set of primers was used to amplify a region of the luciferase gene taken as an internal control (MV3F2 and MV4R2, Supplementary Table S2). Changes in the RL state of the switch were determined by the ΔΔCT method (44). Basal RL levels for *PB LUC:YFP* plant were calculated using control samples agroinfiltrated with P19. Maximum RL levels for the *RL LUC:YFP* plant were calculated with control samples agroinfiltrated with P19. RL levels of the *PB LUC:YFP* plant were normalized to maximum RL levels of *RL LUC:YFP* plant.

### Generation and characterization of *N. benthamiana* transgenic plants

Fully expanded leaves were sterilized and used to obtain 0.5 cm diameter leaf-discs with a cork-borer. After an overnight incubation in co-culture medium Murashige-Skoog supplemented with vitamins (Duchefa), 3% sucrose (Sigma-Aldrich), 0.8% Phytoagar (Duchefa), 1 μg/ml 6-Benzylaminopurine (Sigma-Aldrich), 0.1 μg/ml 1-naphtalenacetic acid (Sigma-Aldrich) the leaf-discs were inoculated for 15 min with the *A. tumefaciens* strain LBA4404 carrying the register module construct (Supplementary Figure S2, Supplementary Table S1). Then, the discs were returned to the co-cultivation medium and incubated for 2 more days in darkness. Next, discs were placed in the selection medium (MS supplemented with vitamins (Duchefa), 3% sucrose (Sigma-Aldrich), 0.8% Phytoagar (Duchefa), 1 μg/ml BAP (Sigma-Aldrich), 0.1 μg/ml 1-naphtalenacetic acid (Sigma-Aldrich), 500 μg/ml carbenicillin, 100 μg/ml kanamycin. Discs were transferred to fresh medium periodically until the callus and then the explants were formed (5–8 weeks). Explants were excised and planted in rooting medium (1/2 MS supplemented with vitamins (Duchefa), 3% sucrose (Sigma-Aldrich), 0.8% Phytoagar (Duchefa), 0.1 μg/ml 1-Naphtalenacetic acid (Sigma-Aldrich), 100 μg/ml kanamycin) until enrooted giving plants considered as transgenic 0 (T0) generation. *In vitro* cultivation was carried out in a long day growth chamber (16 h light/ 8 h dark, 25°C, 60–70% humidity, 250 μmol·m^−2^·s^−1^ photons). Samples from T0 plants for phenotyping were collected once the plants were sufficiently developed to harvest 20 mg of tissue to perform the Fluc and YFP quantifications. The expression of the reporter genes was analyzed as described in the ‘Quantification of reporter gene expression’ section, to select T0 plants for subsequent analysis (Supplementary Figure S3). Selected plants from the T0 generation produced T1 seeds by self-pollination, which were analyzed for segregation of the transgene in kanamycin plates for antibiotic selection of transgenic 1 (T1) seedlings (resistant to kanamycin). After germination, the lines that exhibited a 3:1 ratio (kanamycin resistant versus kanamycin sensitive) were scored as a single copy by the chi-square test. For subsequent experiments, single-copy scored seeds of the T0 lines were germinated in kanamycin plates and then, kanamycin resistant T1 seedlings were transferred to soil to perform the agroinfiltration analysis. Note that T1 plants used in the experiments (selected as being kanamycin resistant) will be two-thirds heterozygous and one third homozygous.

### Generation of *N. benthamiana* transgenic hairy roots

*Agrobacterium rhizogenes* strain 15834 (45) was used to deliver the estradiol-inducible construct and obtain transgenic *N. benthamiana* hairy roots. WT plants were used for transformation of the estradiol-inducible luciferase (EI Luc) construct (Supplementary Table S1). Transgenic *PB LUC:YFP* T1-2 plants were used for the transformation of the estradiol inducible Int (EI Int) construct (Supplementary Table S1). Fully expanded leaves were used to obtain sterilized leaf-discs which were cultivated for one day in co-cultivation medium without hormones. Saturated agrobacterium cultures were sub-cultivated and grown overnight. The culture medium was removed by centrifugation at 4,500 rpm for 15 min, and then the bacterial pellet resuspended and adjusted with Murashige-Skoog medium to an OD600 = 0.3. Those cell cultures were employed for inoculation of the sterilized leaf-discs for 30 min under agitation. Next, the leaf-discs were blotted, placed in co-cultivation plates and incubated in darkness for 2 days. Then, the discs were transferred to selection plates without kanamycin nor hormones. The medium was periodically renewed until roots emerged. Transformed roots were identified using a Leica MacroFluo MZZ16F with a DsRED filter. See ‘Generation and characterization of *N. benthamiana* transgenic plants’ for the culture medium and growth conditions.

### Estradiol induction experiments

Individual DsRED (+) hairy roots transformed with the EI LUC and EI Int constructs were isolated and divided into two with a scalpel and then separated in Murashige-Skoog medium supplemented or not with 20 μM β-estradiol (Sigma-Aldrich). After 3 days, bright field and red fluorescence images were acquired using a FujiFilm LAS-3000 imager. Then, 100 μM luciferin diluted in Murashige-Skoog medium was added, and the roots were incubated for 30 min at room temperature. Chemiluminescence images of roots were then acquired using the same device and the ‘ultra’ mode settings with one second of exposure. Finally, all the roots were transferred to fresh Murashige-Skoog medium without estradiol and grown for 7 days and imaged again. Samples of induced roots were collected and stored at −80°C to perform Fluc/Rluc quantification and PCR-analysis. To this end, approximately 20 mg of ground roots were used. The same protocols for Fluc/Rluc quantification, gDNA extraction, and PCR described in the ‘Quantification of reporter gene expression’ and ‘Genotyping and quantitative PCR analysis’ sections were applied. The growth conditions were as described in ‘Generation and characterization of *N. benthamiana* transgenic plants’.

## RESULTS

### Design of a modular reversible genetic switch for plant systems

We adapted a previously reported strategy used to build synthetic memory (29) to plant systems, by making use of eukaryotic regulatory elements and the bacteriophage ϕC31 integrase (Int) and its cognate RDF (26). This plant genetic switch comprises an invertible DNA promoter element flanked by the Int *attP* and *attB* recombination sites in opposite orientations. This configuration of the switch is referred to as the PB state (named by the presence of the *attP* and *attB* DNA sites) (Figure 2A). In the initial state, the promoter drives the expression of a gene of interest (*GOI2*) positioned downstream of the invertible DNA segment. The orientation of the promoter can be changed by the action of the ϕC31 integrase, which catalyzes recombination between *attP* and *attB* to generate *attR* and *attL*, referred to as the RL state. We refer to this reaction as SET operation. The RL configuration can be recombined back to the PB state by the action of the Int + RDF complex, an operation we refer to as RESET. In the PB state, the promoter in the invertible DNA segment drives transcription of the gene positioned at the right side of the switch (*GOI2*, Figure 2A), whereas in the RL state the gene located at the left of the switch becomes activated (*GOI1*, Figure 2A). When *GOI2* is on, *GOI1* should be off, and vice versa. For clarity, we adopted the convention of naming these DNA constructs *PB GOI1:GOI2* and *RL GOI1:GOI2*, where the state of the switch is denoted by the letters *PB* or *RL*, and the active gene is underlined.

We took advantage of the modularity of the GoldenBraid assembly platform (41) to create a switch that could be easily repurposed for other plant synthetic biology applications. To this end, the genetic switch was structured in three standard and interchangeable DNA parts: (i) the coding sequence to the right of the switch encoding *GOI2*, (ii) the PB or RL invertible elements, and (iii) the coding sequence to the left of the switch encoding *GOI1* (Supplementary Figure S1). The invertible element comprised the strong CaMV35S promoter (*35S*) and a tomato Metallothionein B terminator (*TMtb*) sequence located upstream of the promoter and in opposite orientation to prevent any leaky backward expression (Supplementary Figure S1). The *att* recombination sites were inserted flanking the terminator and promoter elements, carefully positioned in the 5′ untranslated region (5′UTR) of the *35S* promoter, downstream of the TATA box (Supplementary Figure S1) to avoid potential interference with the transcription start site (TSS) that could hinder efficient expression.

For the initial qualitative characterization of this switch in plants, we used the fluorescent reporters yellow fluorescent protein (YFP) and a red fluorescent protein (DsRED) as a readout of the state of the switch. The DNA parts encoding these reporter genes were assembled with the *PB* and *RL* invertible elements to create the *PB DsRED:YFP* and *RL DsRED:YFP* switches (Figure 2). Next, we performed *Agrobacterium-*mediated transient expression tests in *N. benthamiana* leaves by co-infiltrating each switch with a constitutively expressed *Int* gene alone or with a combination of constitutively expressed *Int* and *RDF* genes. Agroinfiltrated-leaves were inspected three days after infiltration by confocal laser microscopy (Figure 2B and C). As anticipated, agroinfiltration of the *PB DsRED:YFP* switch alone yielded bright YFP expression, with no DsRED detected. Co-infiltration of *PB DsRED:YFP* with Int (SET operation) resulted in robust activation of DsRED expression and deactivation of YFP expression in most cells, as shown in Figure 2B. Similarly, agroinfiltration of *RL DsRED:YFP* switch alone produced only DsRED (+) cells, whereas co-expression of *RL DsRED:YFP* + Int + RDF (RESET operation) resulted in activation of YFP expression (Figure 2C). However, we observed an incomplete effect of the RESET operation. After RESET, some cells expressed both YFP and DsRED, and others remained unswitched expressing DsRED only. Both Int and RDF are known to be necessary to catalyze *attR x attL* recombination (26); therefore, co-expression of *RL DsRED:YFP* with Int alone did not result in an observable change in reporter expression (data not shown). Altogether, these results provide a snapshot of the plant-customized invertible switch and demonstrate that the two states can be switched by the SET and RESET operations using either Int or an Int + RDF combination.

### Quantitative characterization of the switch in stably transformed *N. benthamiana* plants

We next set out to evaluate the expression kinetics of the SET and RESET operations in detail (i.e. measure the time-course of activation and deactivation of reporter genes). We assembled four new switch constructs comprising firefly luciferase (*LUC* or Fluc) as a reporter gene: *PB LUC:YFP, PB YFP:LUC, RL LUC:YFP* and *RL YFP:LUC* (Supplementary Figure S2A). Additionally, a constitutively-expressed renilla luciferase (*REN* or Rluc) was assembled to each construct. The activity of both luciferases (Fluc and Rluc) can be measured independently in the same sample and thus, Rluc can be used as an internal reference to compensate for sample variability due to transformation efficiency in transient assays and/or gene dosage and positional effects in stable transformants. The four new versions of the switch were first characterized transiently in wild-type (WT) *N. benthamiana* plants. Changes in the state of the switch were tracked by measuring Fluc activity over constant expression of Rluc (i.e., Fluc/Rluc ratios) over time, starting at 24 hours post-infiltration (hpi) (Supplementary Figure S3). We observed rapid changes in Fluc/Rluc in the switches operated by both SET and RESET operations, demonstrating the suitability of this reporter system to monitor the state of the switch over time. As anticipated, operation of *PB LUC:YFP* and *RL YFP:LUC* resulted in an increase of Fluc/Rluc, while the operation of *PB YFP:LUC* and *RL LUC:YFP* decreased the Fluc/Rluc ratio as compared with non-operated controls (Supplementary Figure S3). Fluc activation was evident as soon as 24 hpi, and the differences were sustained over time.

The experiments described so far enabled quick characterization of the genetic switches by transient expression in WT plants; however, the inability to control the number of switch molecules delivered to each cell via agroinfiltration is a major limitation for the accurate characterization of these genetic elements. Furthermore, the genomic integration of the switches is a necessary step to enable their use in relevant biotechnology applications. For this reason, we set out to stably insert the Fluc switches into the genome of *N. benthamiana* plants to enable a robust characterization of their behavior in plants. First, we assembled the four luciferase switches (*PB LUC:YFP, PB YFP:LUC, RL LUC:YFP* and *RL YFP:LUC*) with a kanamycin selection marker (Supplementary Figure S2B). Then, wild type *N. benthamiana* leaves were transformed using these constructs to obtain transgenic T0 plants, which were initially screened based on YFP and Fluc expression levels (Supplementary Figure S4). Seeds from best performing T0 lines were grown in kanamycin to determine transgene copy number of each line. We selected one line per switch construct containing a single copy transgene: T1-A2 (*PB LUC:YFP*), T1-B5 (*RL LUC:YFP*), T1-C4 (*PB YFP:LUC*) and T1-D20 (*RL YFP:LUC*). These lines were transferred to soil and grown until leaves reached the appropriate size for agroinfiltration.

**Figure 3.**
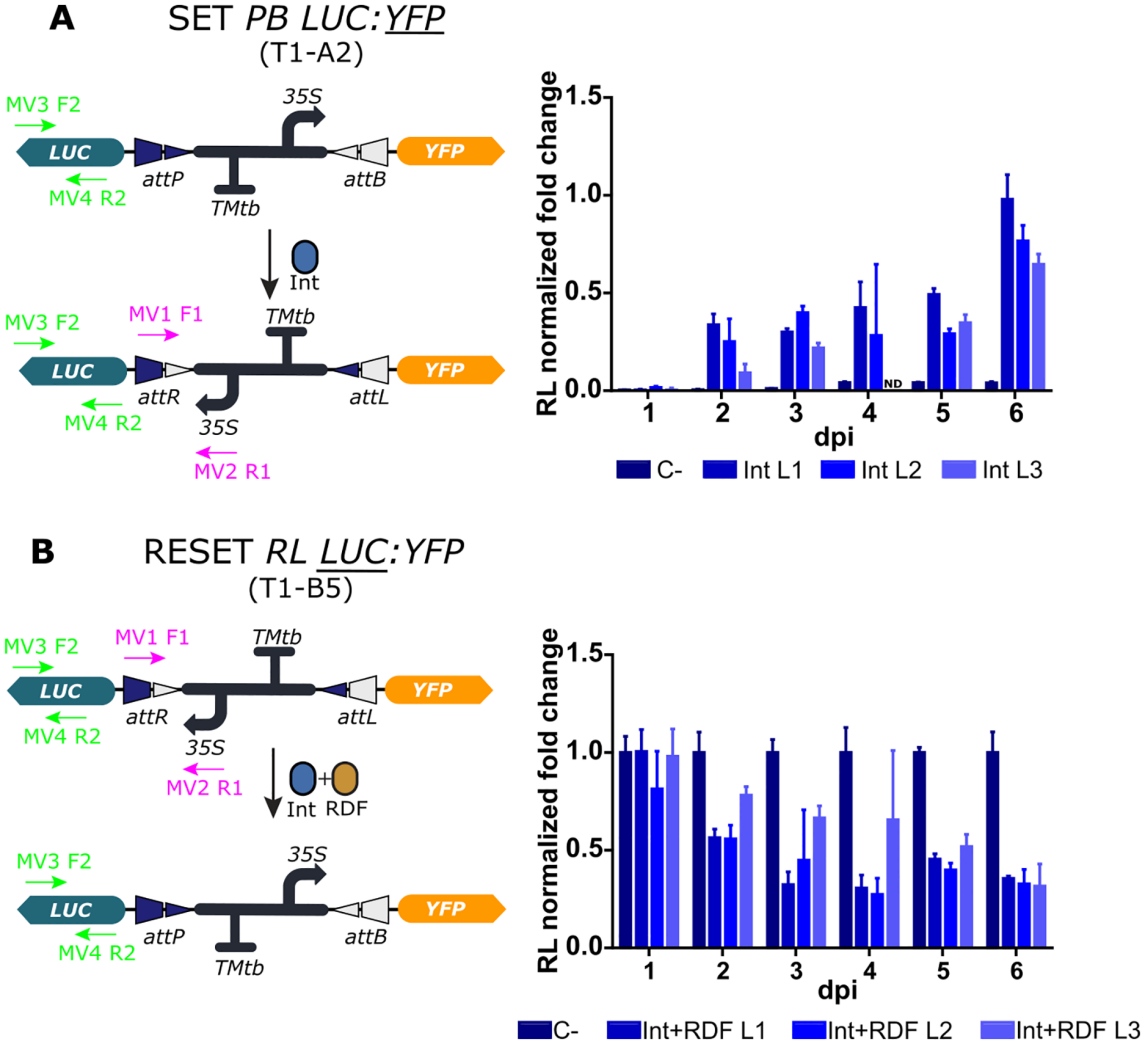
qPCR-based quantitative assessment of switch dynamics in stably transformed *N. benthamiana* plants. (**A**) The SET operation dynamics were monitored up to 6 dpi at the DNA level in three consecutive Int-infiltrated leaves (L1-L3) of the T1-A2 line (*PB LUC:YFP*) by quantifying the increase of the RL state of the switch by qPCR using primers MV1F1 and MV2R1 depicted in the figure. (**B**) The RESET operation was monitored in three (Int+RDF)-infiltrated leaves (L1-L3) of the T1-B5 line (*RL LUC:YFP*) by quantifying the decrease of the RL state of the switch using the same primers as in (A). Each time point comes from a different leaf of the same plant and each data point is normalized to the mean of the control of a different (for the SET *PB LUC:YFP* T1-A2) or the same (for the RESET *RL LUC:YFP* T1-B5) plant. C- indicates controls infiltrated with P19. ND indicates non determined. Values plotted correspond to the mean of three technical replicates ± SD.

We started by analyzing the effect of the switch operations at the DNA level in transgenic plants by qPCR. We designed a specific set of primers that amplified the *attR* site enabling accurate monitoring of increase or decrease of the RL state of the switch after either SET or RESET operations. The line T1-A2 (*PB LUC:YFP*) was used for the analysis of SET, and the line T1-B5 (*RL LUC:YFP*) for the analysis of RESET (Figure 3). The operations were performed as described earlier by *Agrobacterium*-mediated transient delivery of Int or Int + RDF genes respectively at a previously optimized optical density. Optimal levels of the Int and the Int + RDF combination were experimentally determined by titrating the optical density (OD) of the respective *Agrobacterium* cultures, a parameter which is known to correlate with the amount of T-DNA delivered to the transformed cells (11) (Supplementary Figure S5). Three leaves from each plant (L1, L2 and L3), located at correlative positions along the growth axis were analyzed separately to rule out any influence of the leaf developmental stage on the efficiency of the operations. Results are shown as normalized fold changes, using the transgenic line carrying the equivalent un-operated switch as reference for normalization. Thus, a 1.0 value measured in a SET operation in line T1-A2 indicates that the operation reached amplification levels equivalent to those measured in the equivalent, un-operated T1-B5 samples. This additional normalization step was introduced to estimate the degree of completeness of the operation. As observed in Figure 3, both SET and RESET operations were detectable at the DNA level, confirming the functionality of the switch when stably integrated into the plant genome. The recombination effect was detectable at day 2 after agroinfiltration of the operators. The SET operation reached its maximum level at the end of the experimental time course, ranging between 70% and 100% estimated conversion rates depending on the analyzed leaf (Figure 3A). The RESET operation (measured as a reduction of DNA abundance) ended at ∼40% at day 3–4, with no further conversion rates observed in any sample up to day 6 (Figure 3B). The variability among samples made it difficult to draw conclusions on the influence of leaf developmental stages, therefore this variable was not incorporated in subsequent experiments.

**Figure 4.**
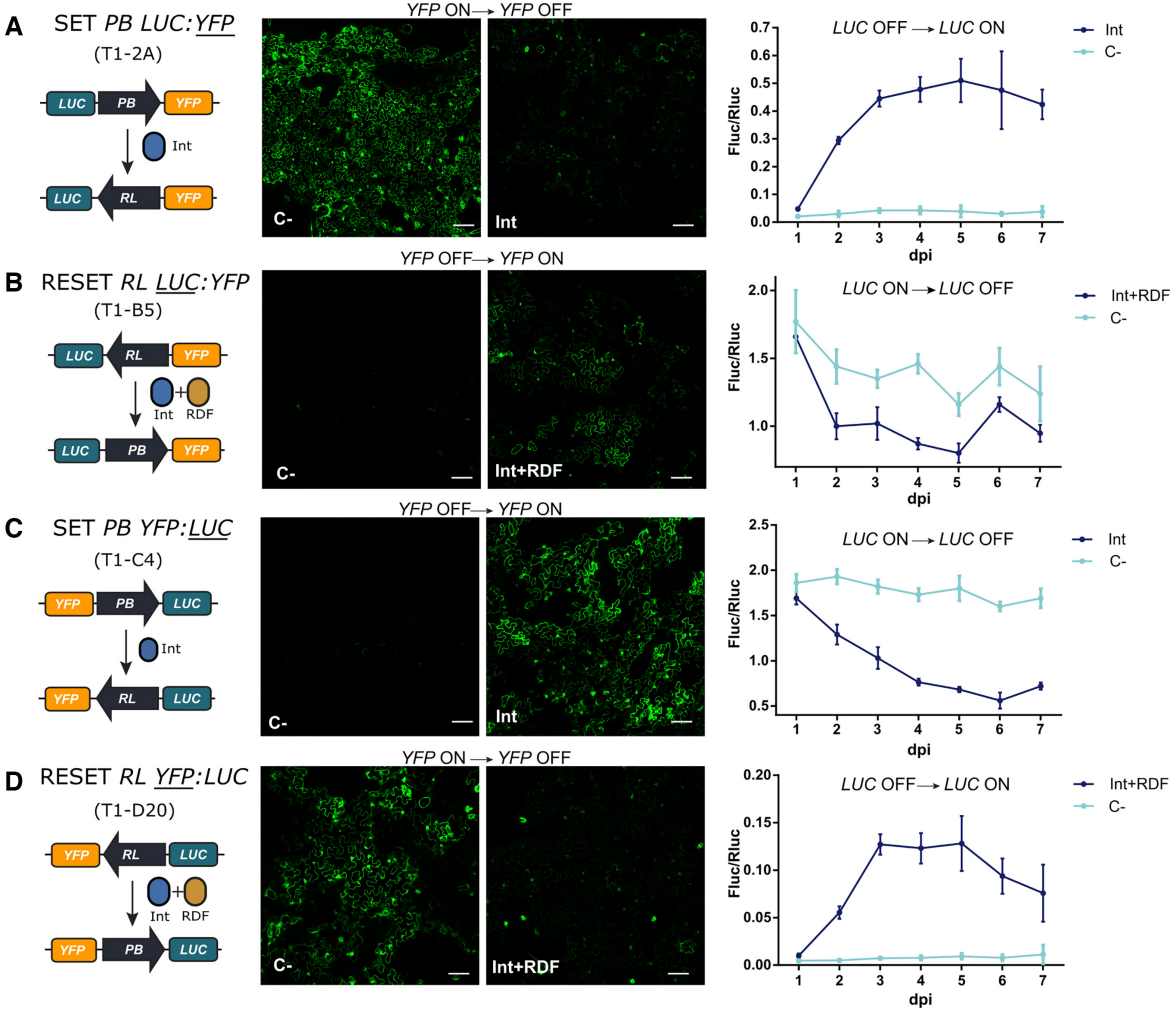
Evaluation of the SET and RESET operations in transgenic *N. benthamiana* leaves. (**A**) SET operation on T1-A2 plants carrying the *PB LUC:YFP* (GB1643) construct. (**B**) RESET operation on T1-B5 transgenic plants carrying *RL LUC:YFP* (GB1645) switch constructs. (**C**) SET operation on T1-C4 plants carrying the *PB YFP:LUC* (GB1644) construct. (**D**) RESET operation on the T1-D20 plant line carrying *RL YFP:LUC* (GB1655) construct. In all samples, respective SET (Int) and RESET (Int + RDF) operations were performed by agroinfiltration. All infiltrations included a construct carrying the silencing suppressor P19. Negative controls (C–) were infiltrated with P19 alone. Left panels show illustrations of each switch operation. Central panels show yellow fluorescence in micrographs taken with a confocal laser microscope. Confocal images represent a tile of nine individual pictures taken 5 days post infiltration; scale bars represent 100 μm. Plots in the right panel represent average Fluc/Rluc values taken every 24 h for 7 days post infiltration (dpi). Experimental points show the mean of normalized Fluc/Rluc values of three agroinfiltrated leaves ± SD. Each experimental point represents a separate T1 plant.

To correlate DNA conversion levels with reporter output, leaves from all four transgenic lines were agroinfiltrated, sampled, and analyzed for Fluc and Rluc expression every 24 h for 7 days (Figure 4); confocal microscopy images were taken in parallel at 72 hpi to also score changes in YFP fluorescence. All four selected T1 lines behaved as expected at the protein expression level when operated by the corresponding effector. Changes in Fluc/Rluc indicating switching started 48 hours post infiltration for both SET and RESET. SET operation on *PB LUC-YFP* line resulted in clear activation of LUC activity after day 2, reaching maximum levels (0.5 relative units) at day 5. This activation correlated with the loss of yellow fluorescence at the cellular level (Figure 4A, Supplementary Figure S6). Normalized Fluc/Rluc levels remained relatively constant in un-switched (control-treated) T1-B5 and T1-C4 lines (those starting with LUC in ON position), whereas switching operations in both lines resulted in a significant reduction of luminescence levels, accompanied by the onset of yellow fluorescence. Note, however, that SET operation in *PB YFP:LUC* line showed a steeper reduction in LUC levels (from **∼**2.0 to 0.5 units) than RESET on *RL LUC:YFP* (from **∼**1.8 to 1.0), (compare Figure 4B and C). Also, RESET of *RL YFP:LUC* turned on LUC activity, but to a lower extent to that observed in the equivalent SET operation (compare Figure 4A and D). In general, SET reactions were shown to be more effective than their RESET counterparts, as previously observed at the DNA level. None of the operations seemed to reach the activity levels of the equivalent un-switched plants, suggesting that conversions are only partial. However, conclusions based on comparisons between plant lines need to be taken carefully due to possible transgene positional effects.

**Figure 5.**
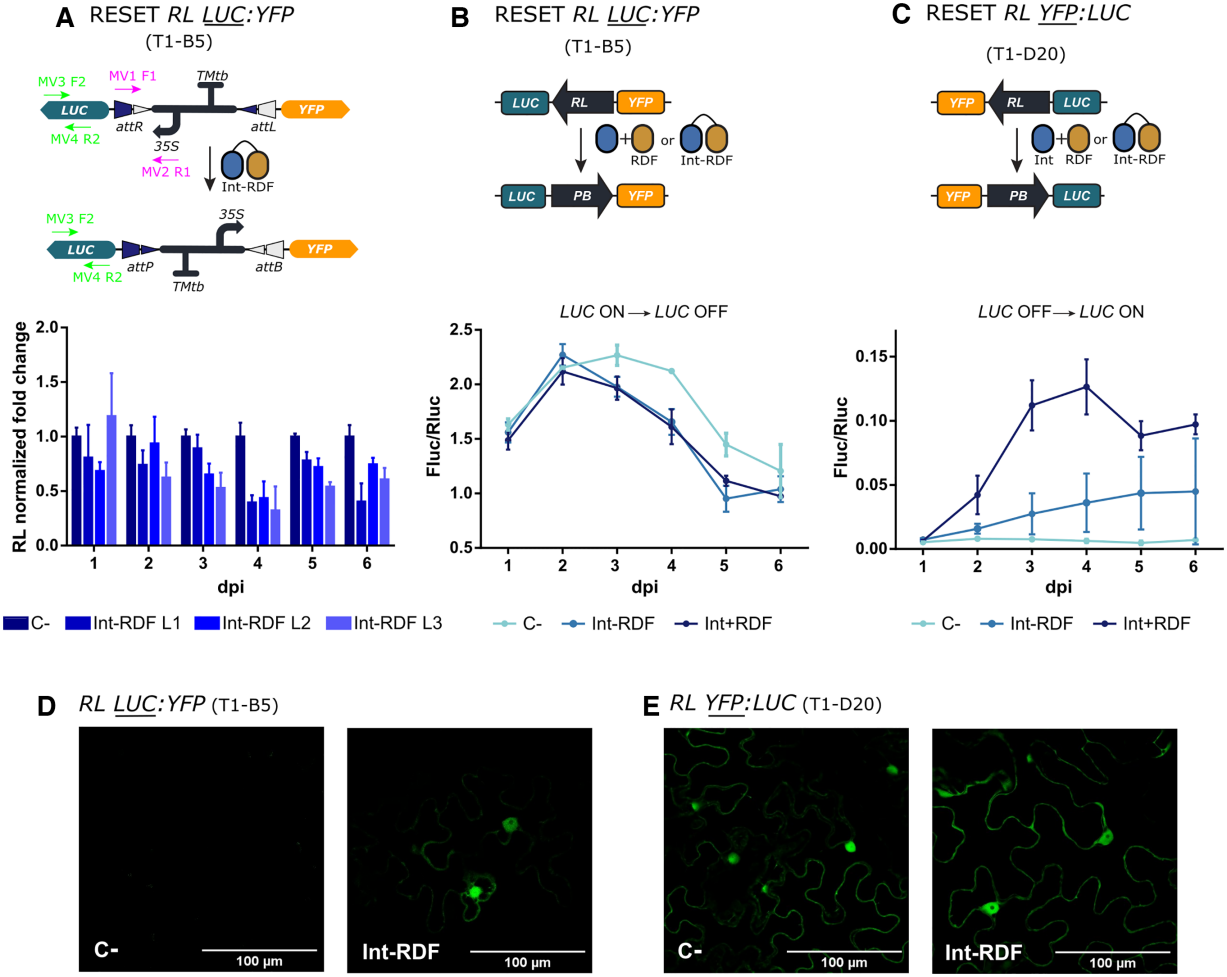
Efficiency of the RESET operation mediated by a Int-RDF fusion protein. (**A**) Analysis of the RESET operation performed by the Int-RDF translational fusion protein by qPCR amplification of the RL state of the switch. The upper panel shows a depiction of the switch operation and the primers used for qPCR amplification. Three (Int-RDF)-infiltrated leaves of the T1-B5 transgenic line (*RL LUC:YFP*) were analyzed as described in Figure 3. (**B**) RESET Fluc/Rluc time course showing the decrease of luciferase activity in leaves of T1-B5 (*RL LUC:YFP*) plants infiltrated with either a P19 control culture (C-, light blue), a Int + RDF mixture (strong blue) or the Int-RDF fusion protein (GB2893, medium blue). (**C**) RESET time course in line T1-D20 (*RL YFP:LUC*) performed by either the Int+RDF mixture or the Int-RDF fusion protein shows the increase in Fluc/Rluc as compared with P19-infiltrated control (C–). (**D**) Confocal imaging showing the activation of the YFP reporter in (Int-RDF)-infiltrated leaves of T1-B5 plant leaf at 5 dpi as compared with a P19-infiltrated negative control. This activation runs in parallel to the deactivation of Fluc activity shown in (B). In (**E**), the YFP signal of the *RL YFP:LUC* in T1-D20 line shows little variations with RESET operation using Int-RDF fusion, evidencing the low activity of the fusion also observed in (C). Experimental points in Fluc/Rluc plots show the mean of normalized Fluc/Rluc values of three agroinfiltrated leaves ± SD.

The results in Figure 4 also showed that, whereas Fluc levels were sustained over time for the SET operation, RESET operated switches showed Fluc/Rluc fluctuations after 5 days post infiltration (dpi). This could be simply due to tissue damage after agroinfiltration, but also could be caused by an excess of Int not bound to RDF, which would cause multiple RESET/SET recombination cycles thus decreasing the stability of the measurements. It has been demonstrated that optimal concentrations of Int and RDF (1:1 or excess of RDF) are necessary to maximize the efficiency of the RESET operation (29,46). Therefore, fluctuations in the Int/RDF expression ratios could make the RESET operation unstable. Besides, the requirement for dual expression also limits the operativity of the switch in practical terms. This prompted us to explore, as a possible solution, the expression of an Int-RDF translational fusion to perform the RESET operation, as described by Olorunniji *et al.*, (47). This fusion would ensure a 1:1 expression ratio of Int:RDF, thus, potentially maximizing the efficiency of the RESET operation. We tested the activity of the Int-RDF fusion by qPCR analysis of the state of the switch, Fluc/Rluc activity and confocal microscopy. Results in Figure 5 show that the Int-RDF fusion was able to promote RESET operation as evidenced by the reduction in *RL LUC:YFP* amplicon quantification, the changes in LUC activity (both in *RL LUC:YFP* and *RL YFP:LUC* conformations), as well as by the activation of YPF reporter. However, in all three cases, the efficiency of the Int-RDF fusion was significantly lower than that observed when the two factors were expressed separately. Further optimizations would be required to ensure the bi-stability and operativity of the switch without severely affecting its efficiency.

### Heritable and reversible memory storage over a full SET/RESET cycle in whole plants

**Figure 6.**
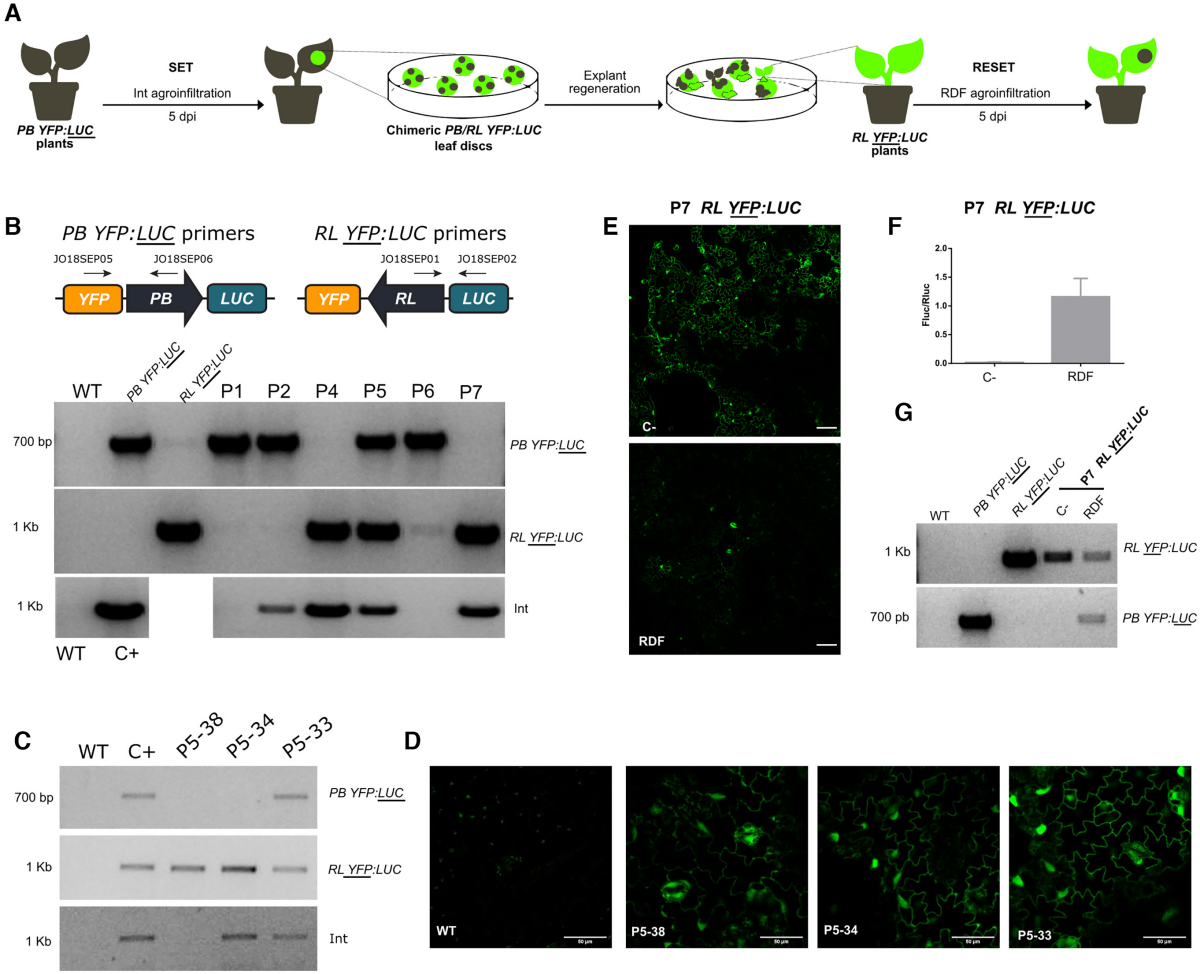
Full SET/RESET cycle of the switch through plant transformation and regeneration process. (**A**) Representation of the experiment conducted to assess the heritability and reversibility of the switch in *N. benthamiana PB YFP:LUC* transgenic lines. *PB YFP:LUC* plants were first agroinfiltrated with Int (GB1531) to catalyze a SET operation that yielded *RL YFP:LUC* switch as a result. Fluorescent leaf discs were collected 5 days post infiltration, sterilized and then cultivated *in vitro* for explant regeneration. The genotype of the explants was then analyzed by PCR. *RL YFP:LUC* plants constitutively expressing Int (e.g. plant #7, P7) were then agroinfiltrated with RDF to RESET to the original *PB YFP:LUC* configuration and demonstrate the reversibility of the switch. (**B**) Agarose electrophoresis gels showing the genotyping results of the different regenerated plants (P1, P2, P4-P7). Two pairs of primers were used to distinguish between the PB and RL configurations as shown in the illustration. The upper panel shows the amplification of the original *PB YFP:LUC* configuration, the middle panel shows the resulting *RL YFP:LUC* state after the SET operation and the lower panel shows amplification results of integrated *Int*. WT genomic DNA was used as negative control while genomic DNAs of the respective transgenic lines were used for positive controls. (**C**) Agarose electrophoresis gel showing the genotyping results for the RL and PB conformations and *Int* integration in representative siblings in the progeny of regenerated plant #5 (P5); genotypes analyzed following the same procedure as in (B). As shown, P5-38 retains the switched *RL YFP:LUC* state in the absence of *Int*. (**D**) Confocal laser microscopy images of the same P5 progeny individuals genotyped in (C). Scale bars represent 50 μm. (**E**) Confocal laser microscopy images at 5 dpi of P7 leaves agroinfiltrated with RDF in presence of P19 (RESET) as compared with a negative control agroinfiltrated with P19 alone. Scale bars represent 100 μm. (**F**) Fluc/Rluc ratio of the same samples as in (E). Bars represent mean Fluc/Rluc values for three different agroinfiltrated leaves ± SD. (**G**) Genotyping results of the same samples as in (E) and (F). WT, *PB YFP:LUC* and *RL YFP:LUC* are the same controls used in (B) and (C).

A distinctive feature of an Integrase-based memory device is the long-term stability of modifications based on DNA recombination (48). Our primary goal was to create a switch that could alternate between two different configurations and whose memory status would be transmitted to the progeny. To this end, we set out to test the memory and reversibility of the switch by performing a full SET/RESET cycle in *N. benthamiana* lines with the switches integrated into the genome. As successive agroinfiltrations of the Int or Int + RDF would result in excessive damage to the plants, we decided to generate calli from leaves after the first SET operation, and then to perform the second RESET operation on fully regenerated plants. We performed the SET or RESET operations by agroinfiltration (instead of classical co-cultivation used in stable transformation) to increase the initial population of switched cells. Therefore, we conducted the experiment illustrated in Figure 6A, using the T1-C4 (*PB YFP:LUC*) stable line to perform a SET operation by agroinfiltration of Int in the leaf. Then, at 5 dpi when the recombination peak was reached, fluorescent discs were excised and regenerated *in vitro* to obtain stably switched calli. We observed both homogeneously switched and unswitched calli, and also chimeric calli made of a mixed population of switched (YFP +) and unswitched (YFP -) cells, probably resulting from incomplete and/or late SET operations taking place after initial cell divisions. Chimeras often appear in organogenesis-based plant transformations when plants regenerate from a mixed population of T-DNA-transformed and non-transformed cells. Note that in our case the observed YFP chimerism reflects the status of the switch, rather than the presence or absence of a genome-integrated T-DNA containing the *Int* transgene. We were able to regenerate seven independent plant lines (P1- P7), one of which (P3) was lost in the process. To assess the configuration of the switch in each line (either the initial *PB YFP:LUC* or the switched *RL YFP:LUC* state), regenerated plants were genotyped using specific primer pairs for PB or RL state. Agroinfiltration of the SET operation could cause unwanted integration of *Int* into the genome, therefore, we also analyzed the integration of *Int* in the same lines by PCR (Figure 6B). Plants P1 and P2 did not show evidence of switching events (only PB state detected), and in P6 only a faint RL band was detected. P1 and P6 showed no presence of *Int*, while P2 showed a weak band. P5 was apparently a chimeric plant in which both switch configurations coexisted. Finally, P4 and P7 were positive only for RL, indicating complete switching to the RL configuration. In all three switched plants (P4, P5, and P7), RL status was accompanied by the presence of *Int* copies stably integrated into the genome. This raised the question of whether the presence of Int was required to maintain the RL status, something that would contradict the memory feature of the system. To rule out this possibility, we screened the offspring of self-pollinated P5 in search of plants that had segregated out *Int*, while maintaining the switched *RL YFP:LUC* status. In our experience, agroinfiltrated leaf disks are prone to multiple T-DNA integration that makes the segregation process extremely arduous. Therefore, we chose P5 for this segregation effort assuming that a chimeric plant had probably resulted from transformation events generating lower transgene copy numbers. Indeed, it required the analysis of 74 segregating T1 plants to finally obtain a single plant (P5-38) showing the expected *Int* (−) / *RL YFP:LUC* genotype (Figure 6C). As expected, P5-38 was also YFP (+) (Figure 6D). Other *Int* (+) plants recovered in the screening contained RL or RL/PB mixed configurations (Figure 6C and D). Altogether, these results demonstrate that the RL state resulting from a SET operation was stably inherited in the absence of *Int*.

We then chose a fully-switched plant (P7) to test the reversibility of the genome-integrated device. The plant was transferred to soil and grown until leaves reached the appropriate size for agroinfiltration. Since P7 was constitutively expressing Int, it only required the agroinfiltration of RDF to perform the RESET operation, thus reversing the state of the switch to the original PB state. P7 leaves were agroinfiltrated with RDF or with a negative control plasmid and analyzed for YFP (Figure 6E) and Fluc expression (Figure 6F). YFP decreased at 5 dpi in RDF samples (Figure 6E) and Fluc activity increased 58-fold over the plant P7 control levels, reaching approximately 60% of the Fluc/Rluc ratios typically observed in the original *PB YFP:LUC* plant (Figure 6F). These results were also genotypically confirmed by PCR-amplification of the switched tissue, as compared to control samples (Figure 6G). Altogether, these experiments demonstrate that the switch, once integrated into the genome can be effectively used to perform a full cycle of SET/RESET operations, and can transmit the memory of its state to the progeny.

### Control of switching by chemical induction of Int

**Figure 7.**
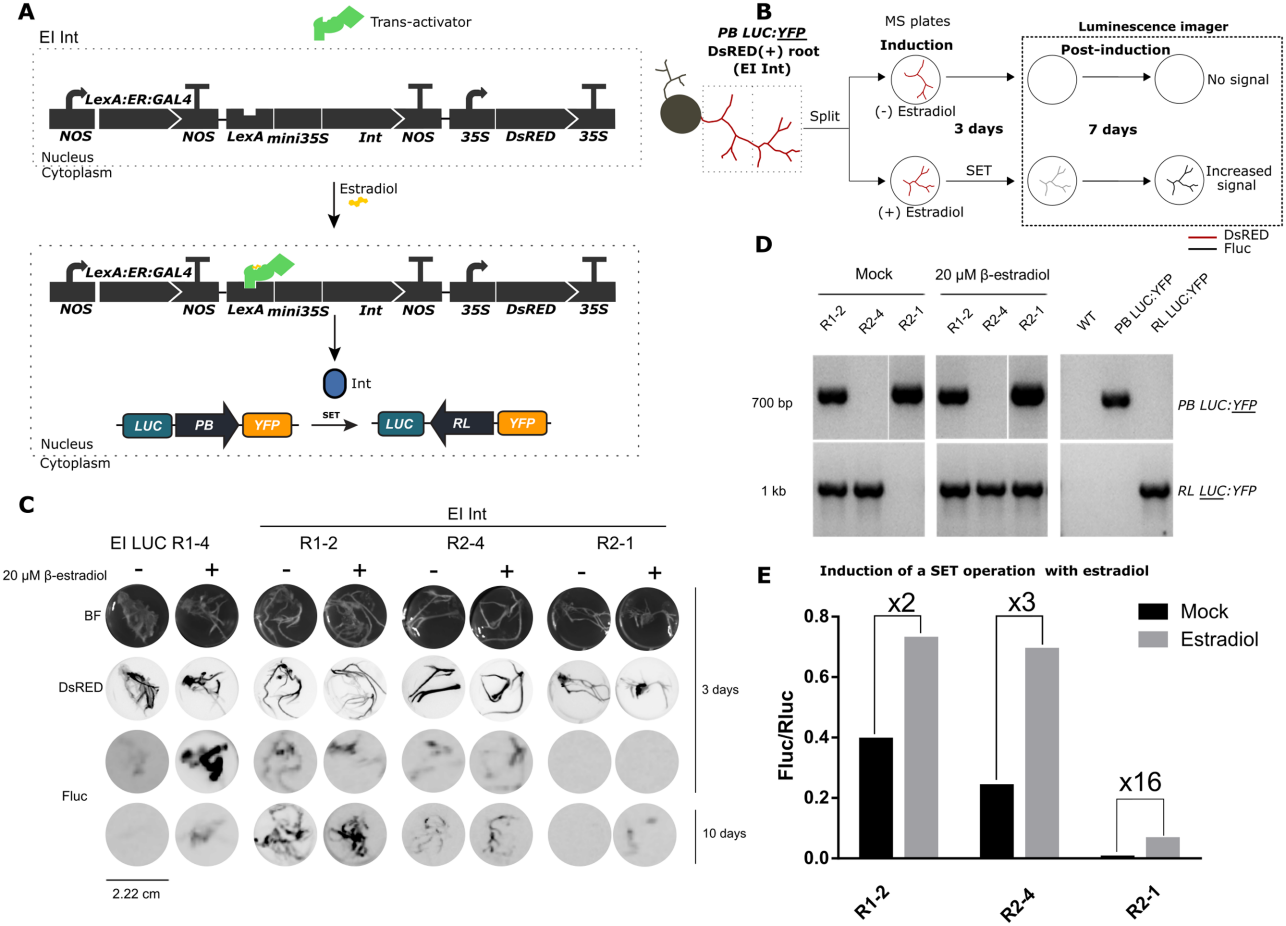
Chemical induction of the Int in stable *N. benthamiana PB LUC:YFP* T1-A2 hairy roots. (**A**) Representation of the estradiol-inducible (EI) system EI Int (GB2313). In the absence of estradiol, the constitutively expressed chimeric trans-activator is confined to the cytoplasm. Upon addition of estradiol, it localizes to the nucleus where it induces the expression of Int. This enables the SET operation of the *PB LUC:YFP* switch turning on Fluc *LUC* expression. (**B**) Diagram of the EI Int experiment in hairy roots. DsRED (+) *N. benthamiana PB LUC:YFP* hairy roots transformed with EI Int were divided and incubated in Murashige-Skoog plates in the presence (SET) or absence of estradiol (Mock) for 3 days. After chemiluminescence imaging, roots were transferred to new estradiol-free plates where they remained for 7 days before imaging them again to measure Fluc activity. (**C**) Images of individual hairy roots samples derived from a T1-A2 plant transformed with EI LUC (GB2388) or EI Int (GB2313) constructs. The first row shows bright-field images (BF); the second row shows red fluorescence of the same samples for the detection of DsRED marker; the third and fourth rows show chemiluminescence images (FLuc) of the same samples taken with a LAS3000 imager at 3 and 10 days after estradiol treatment respectively; (**D**) Genotyping results of uninduced (left panel) and induced (central panel) EI Int hairy roots, including WT, *PB LUC:YFP* and *RL LUC:YFP* gDNAs as controls (right panel). A specific pair of primers for *PB LUC:YFP* and *RL LUC:YFP* amplification was used. (**E**) Quantification of the Fluc/Rluc ratios of estradiol-induced and mock EI Int hairy root lines. Roots were ground and 20 mg of powder was analyzed. Bars represent the mean of three technical replicates for an individual root.

The experiments described so far used agroinfiltration to deliver Int or the Int + RDF combination, a strategy that does not allow precise spatiotemporal control of expression. We sought to achieve more operative control over the recombination process by coupling the expression of Int to an estradiol-inducible system, which was named estradiol-inducible Int (EI Int). To this end, a new genetic module was assembled encompassing three transcriptional units encoding (i) a chimeric trans-activator (*LexABD* (LexA binding domain)::*ER* (estradiol receptor)::*GAL4AD* (GAL4 activation domain); (ii) an inducible Int with the *LexA* operator and the *mini35S* promoter; and (iii) a DsRED fluorescent marker of successful transformation (Figure 7A). In this system, the trans-activator is constitutively expressed and remains in the cytoplasm. Upon addition of estradiol, the trans-activator translocates to the nucleus where it binds the DNA operator, inducing the expression of Int. Expression of Int should result in a SET operation of the stably transformed switch, which is evidenced by the activation of Fluc luminescence.

We used *A. rhizogenes* to stably super-transform the EI Int system in a kanamycin-resistant transgenic plant of the T1-A2 line, which carried a single copy of *PB LUC:YFP* construct. This transformation system induces the generation of hairy roots (each root is an individual transformation event), which is an ideal system for high-throughput chemical screening. We performed the estradiol-induction (EI) experiments as shown in Figure 7B. Successfully transformed (DsRED +) roots were incubated with or without estradiol for 3 days and evaluated for Fluc expression by luminescence image analysis. Subsequently, the roots were transferred to fresh estradiol-free Murashige-Skoog plates and incubated for seven additional days (a total of 10 days), when the second round of luminescence imaging was recorded. In parallel, WT *N. benthamiana* plants were also transformed with a construct bearing an estradiol-inducible Fluc reporter (EI LUC) as a positive control device. As shown in Figure 7C and Supplementary Figure S7, estradiol-induced EI LUC hairy roots showed higher luciferase signal than controls at 3 days after estradiol treatment, but most of this signal was lost at 10 days after treatment. On the other hand, EI Int root lines maintained Fluc activity over time up to 10 days after treatment. Unexpectedly, most EI Int hairy root lines (represented here as lines R1-2 and R2-4) showed a high Fluc background in the absence of estradiol, probably due to leaky expression of the inducible construct. Only the R2-1 line displayed a clean background, and although Fluc intensity at 3 days after estradiol treatment was almost undetectable, it reached moderate levels at 10 days after treatment (Figure 7C). The diagnostic PCR-amplification of the *PB LUC:YFP* and *RL LUC:YFP* configurations of the different samples at 10 days after estradiol treatment (Figure 7D) also confirms this interpretation. R1-2 and R2-4 showed *RL LUC:YFP* amplification with and without estradiol induction, confirming that the unwanted switching had occurred even in the absence of the hormone signal. Importantly, R2-1 showed no *RL LUC:YFP* amplification in mock treatment, but a strong signal upon induction with estradiol. This result confirmed our hypothesis that the observed increase in Fluc activity in R2-1 at 10 days after treatment was due to a successful estradiol-inducible SET operation of the switch. As additional confirmation, we quantified the luminescence at day 10 comparing induced and non-induced roots for each line (Figure 7E). R1-2 and R2-4 lines showed a moderate 2–3-fold Fluc/Rluc increase upon chemical induction, probably resulting from a more accelerated switching in the presence of estradiol as compared with the untreated mock. In contrast, R2-1 showed substantially lower Fluc/Rluc ratios but much stronger induction (16-fold) when compared to their leaky counterparts. In conclusion, these results show that chemically-inducible Int can control the activation of a genetic switch. However, further fine-tuning of the chemical induction system is required to avoid the leaky expression of the Int. A number of factors including the genomic localization of the inducible Int or its copy number could result in basal or sporadic expression, resulting in unwanted switching.

## DISCUSSION

In this work, we sought to expand the molecular toolbox for plant synthetic biology by designing a reversible memory switch for whole plants. Currently, the repertoire of devices to build synthetic memory in plants is limited, being a red-light controlled memory switch for protoplasts the only case reported (22). While this system provides great spatiotemporal resolution of gene regulation, its adaptation to full plants remains unrealized due to two main challenges: (i) it requires strict light conditions, which interfere with natural plant development and preclude its application in the field, and (ii) it only enables short-term memory, which prevents its use in applications that require long-term and inheritable memory storage. On the other hand, serine integrases are powerful molecular tools that catalyze a stable change in the DNA, thus enabling long-term and heritable data storage that is not affected by environmental factors or protein degradation. As a result, integrases have been extensively used for many synthetic biology applications, including the design of memory switches for bacteria and mammalian cells (49). We adapted the previously described bacterial memory switch by Bonnet *et al.*, which was based on the Bxb1 phage integration system (29). This switch enabled transcriptional control of two reporter genes by inversion of a promoter element whose orientation was defined by the action of the Bxb1 integrase and the Bxb1 excisionase. To optimize the adaptation of this system to plants we decided to use the bacteriophage ϕC31 integrase and RDF because this integrase has been shown to be highly active in eukaryotic organisms (50,51) and has been widely used for genetic engineering in plants (52). The architecture of the switch was designed to be fully modular following the GoldenBraid assembly standard (11,41), so it could be easily adapted to multiple plant synthetic biology applications by just performing a single-step assembly reaction (see ‘Supplementary methods’ section).

Our first objective was to fully characterize the behavior of this new memory switch in *N. benthamiana* plants. To do so, we built several constructs with the switch in different starting conformations (PB or RL) and different reporters. This enabled us to independently test the efficacy of each reaction for the first time in plant systems: SET (*attP* x *attB* reaction) and RESET (*attR* x *attL* reaction). We used fluorescent and luminescent proteins as reporters to evaluate the activation state and kinetics of the switch. First, the switches were tested transiently by agroinfiltration. In the initial state, both PB and RL conformations showed strong expression of the active reporter and no noticeable expression of the inactive reporter, indicating that the design of the genetic switch successfully avoided leaky expression (Figure 2 and Supplementary Figure S3). Co-delivery of PB switches + Int (SET), or RL switches + Int + RDF (RESET) via agroinfiltration demonstrated that both operations were functional, as shown by activation of the previously inactive gene (Figure 2 and Supplementary Figure S3). These results confirmed the activity and reversibility of the switch in plants. To demonstrate the applicability of this tool for long-term memory storage, we also studied the functionality of the switch when stably integrated into the plant genome. Quantitative PCR analysis of the SET operation in transgenic lines with PB integrated into their genome showed that it is possible to achieve an almost complete (>80%) conversion of the switch after six days of Int transient expression (Figure 3A). Reporter activation analysis in transgenic plants also demonstrated the high efficiency of the SET operation (Figure 4A). In contrast, quantification of RESET operations yielded only moderate conversion levels, reaching a maximum of 60% conversion after six days (Figure 3B). Incomplete switch operations were also evident when reactions were monitored using expression of Fluc activity or YFP fluorescence. (Figure 4B), although in these cases, switch conversion rates can be underestimated due to the slow decay of the reporters and the possible interference on transcription levels exerted by Int and RDF factors binding the recombination sites (11). Differences in switch copy numbers in segregating T1 plants (carrying one or two copies per cell), could also have had an influence in the time course dynamics and completion levels; however we believe that this is fairly compensated by the normalization carried on in qPCR experiments and luciferase assays against a reference sequence/gene in the same T-DNA.

Although a uni-directional memory switch for plants is in itself an extremely useful tool, a reliable reversible switch would translate into a more expanded range of applications. Further optimization of the conversion efficiencies, especially for RESET operations, will be needed to ensure the bi-stability of the switch. Phage RDFs have been studied due to their ability to reverse the directionality of a site-specific recombination reaction via direct interaction with the cognate integrase, i.e. to catalyze *attR x attL* reaction (26,53). However, this reaction has been shown to be highly inefficient when the stoichiometry of the integrase and RDF is not optimal (i.e. less RDF than Int) (29). Therefore, these authors tested several combinations of genetic constructs to increase the RDF expression levels and reduce the half-life of the integrase achieving a unidirectional reaction with 90% efficiency. We followed a similar approach by increasing RDF expression levels through the *CaMV35S* promoter, which has about 10-fold higher expression than the Nopaline Synthase promoter used to express the Int (54). Additionally, we optimized the optical density of the *A. tumefaciens* cultures during transient expression to optimize the efficiency of the RESET operation (Supplementary Figure S5). However, we still saw incomplete *attR x attL* recombination of all induced cells, as seen by fluorescent imaging of leaf cells (Figure 2C) and transformed calluses. Recently, Olorunniji *et al.*, 2017 reported higher *attR x attL* recombination efficiency by creating a fusion of Int to RDF (47). We reasoned that using this new Int-RDF fusion as the RESET operator in our plant switch could help to solve bidirectionally issues. Furthermore, by fusing Int and RDF in a single transcriptional unit that governs RESET operations, the operativity of the switch and its connection to upstream regulators would be considerably simplified. The Int-RDF fusion tested in this work was functional but showed reduced activity levels when compared to the two proteins expressed individually (Figure 5). A potential solution to this issue could be to couple the expression of the RDF to a delay circuit, as recently described by Zhao *et al.* (28). These authors created a single-input binary counter by coupling the expression of RDF and a transcriptional repressor to one state of the switch (instead of being externally induced), so that the reversibility of the switch was controlled by a single input of Int. This approach enabled the repeated operation of the switch in SET/RESET/SET cycles; however, they still reported incomplete RL-PB conversion. A recent study exploring the mechanism of the ϕC31 integrase and RDF suggests that optimal stoichiometry between Int and RDF is not sufficient to achieve maximum efficiency of the *attR* x *attL* reaction, instead, their mathematical model suggests that the equilibrium constants of recombination would need to be modified (46). Further understanding of the structural mechanism of serine integrases (46,55) and protein engineering efforts (47,56) are necessary to fully realize their potential and their application for fully reversible genetic switches.

After individual characterization of the SET and RESET operations, we decided to test the performance of a full SET/RESET cycle to evaluate the memory of the switch. However, a drawback of the agroinfiltration method is the cellular damage produced by the bacteria during the process, especially after repeated injections on the same leaf. Instead, we decided to regenerate plants after agroinfiltration of Int (SET), to then agroinfiltrate them with Int + RDF (RESET) when fully regenerated. We induced SET in a stable *PB YFP:LUC* plant, and then regenerated *in vitro* the resulting fully switched *RL YFP:LUC* plants (Figure 6). One out of three regenerated plants showed complete switching into *RL YFP:LUC* state and constitutive expression of YFP. We observed that some regenerated plants had stably integrated the *Int* transcriptional unit after agroinfiltration, but we demonstrated that the Int transgene could be sexually segregated from the *RL YFP:LUC* state. This result proved that the switch is able to register the SET operation at the DNA level in the absence of integrase, thus keeping long-term memory even after full plant regeneration. Subsequently, we induced RESET on the newly generated *RL YFP:LUC* P7 plant, which showed an increase of Fluc expression and decrease of YFP (Figure 6E). This demonstrated that P7 cells returned to the original *PB YFP:LUC*, showing a successful SET/RESET cycle over the same switch integrated into the genome. However, we observed incomplete efficiency of the RESET operation, and further optimizations will need to be done to overcome this limitation. Presumably, these changes at the DNA level should be heritable, and be transmitted to the progeny as other authors reported in *Arabidopsis thaliana* for Bxb1 (57) and ϕC31 (52).

Finally, we coupled the expression of Int to an estradiol-inducible system, as a proof of concept of externally inducing the SET operation to control the switch in *N. benthamiana* hairy roots (Figure 7). Unfortunately, several transgenic root lines showed unwanted activation of the switch due to leaky expression of the integrase. Chemically-inducible systems with low basal expression have been reported in plants (14–18); however most of these systems were tested in *Arabidopsis*, and it is possible that chassis-specific conditions (e.g. phytosterol contents) could affect background expression levels. Basal leakiness should be taken into consideration in the design of complex circuits based on integrases, as even low basal expression levels can cause unwanted inactivation of the switch. Regulation of site-specific DNA excision has been previously reported with the tyrosine recombinase Cre*/loxP* system in *A. thaliana* using an estrogen-based chemically-inducible system (XVE induction system) (58). In this study, the authors counter-selected the leaky transformation events by flanking the inducible recombinase and the selection marker with the *loxP* sites. If leaky, the recombinase would excise this cassette and therefore the transformants would be lost in a selection media. In this work we did not apply negative selection pressure, so all the transformation events were kept. We were able to recover one root line with low basal expression but still switchable upon estradiol induction (Figure 7E). After activation, low but sustained expression of the Fluc reporter was observed. Similar results were obtained by Hoff *et al.* when using a heat-shock regulated Cre/*loxP* (59). They found the system was leaky but still inducible, and that induction worked better in lines with low reporter gene expression as in our case. In order to circumvent this issue, different strategies could be implemented, such as the use of degradation tags to regulate the half-life of the integrase or an induction system relying on logic gates to regulate the expression of the operators. In this regard, a split ϕC31 integrase that can be reconstituted after *trans*-splicing of both components has been developed recently (56).

Summarizing, in this work we created the first modular memory switch for plants making use of the bacteriophage ϕC31 integration system. This switch was reversible and had memory, although future efforts are needed to optimize the RESET reaction and avoid unwanted activation. Even though the complexity of big multicellular organisms can be a challenge for extensive characterization of new molecular tools, new advances in the field will allow harnessing their potential. We expect that this system will provide a new tool for plant synthetic biology that will enable engineering plants with more complex gene networks and circuits.

## Supplementary Material

gkaa104_Supplemental_Files

## Notes

Present address: Alfredo Quijano-Rubio, Institute for Protein Design, University of Washington, Seattle, WA, USA.
