# Supplementary material for: A memory switch for plant synthetic biology based on the phage ϕC31 integration system
Source: Nucleic Acids Res. 2020 Feb 21;48(6):3379–94. doi: 10.1093/nar/gkaa104 (PMC7102980; doi:10.1093/nar/gkaa104)

Supplementary figures

Figure S1: Architecture of the genetic memory switch. The switch is structured in three DNA parts (i) reverse coding sequence (CDS) or gene of interest 1 (GOI1), (ii) an invertible element that is the PB or RL states of the switch and (iii) a forward CDS or gene of interest 2 (GOI2). The genes of interest (GOI) are cloned as individual parts in Level 0 GoldenBraid cloning schema, using the depicted overhangs and then assembled with the PB or RL invertible elements to create a Level 1 construct for the reversible regulation of each GOI. A detailed view of this process is given in the Supplementary Materials and Methods section.

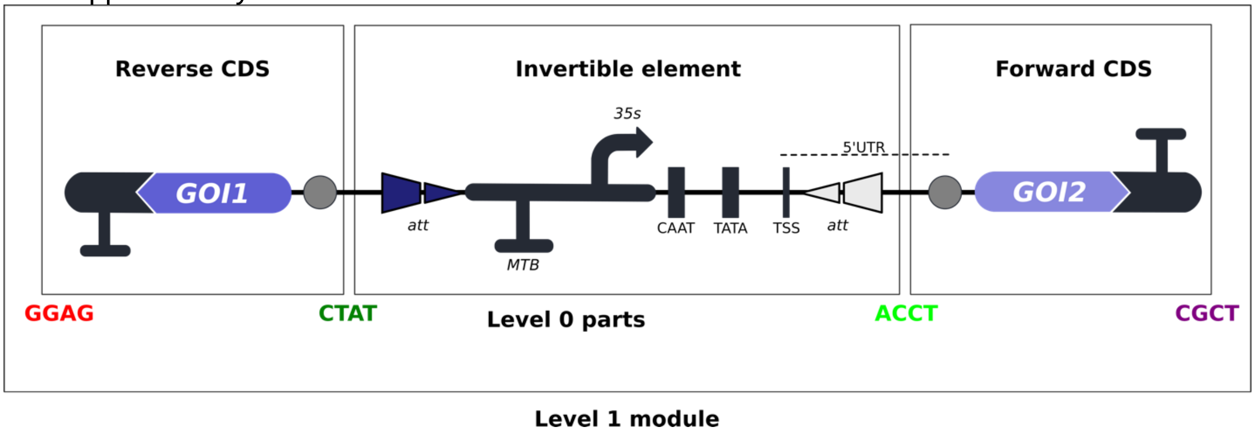

Figure S2: DNA constructs used in the transient expression experiments of Fig. S3 **(A)** and for the generation of transgenic lines in Fig. 3 and Fig 4 **(B)**. Constructs comprise (i) the different versions of the switch with *YFP* and *LUC* as reporter genes, (ii) a transcriptional unit for the constitutive expression of renilla luciferase (Rluc, *REN*), (iii) and a transcriptional unit for the constitutive expression of the silencing suppressor P19 for transient expression experiments or the kanamycin selection marker for stable plant transformation (*NptII*).

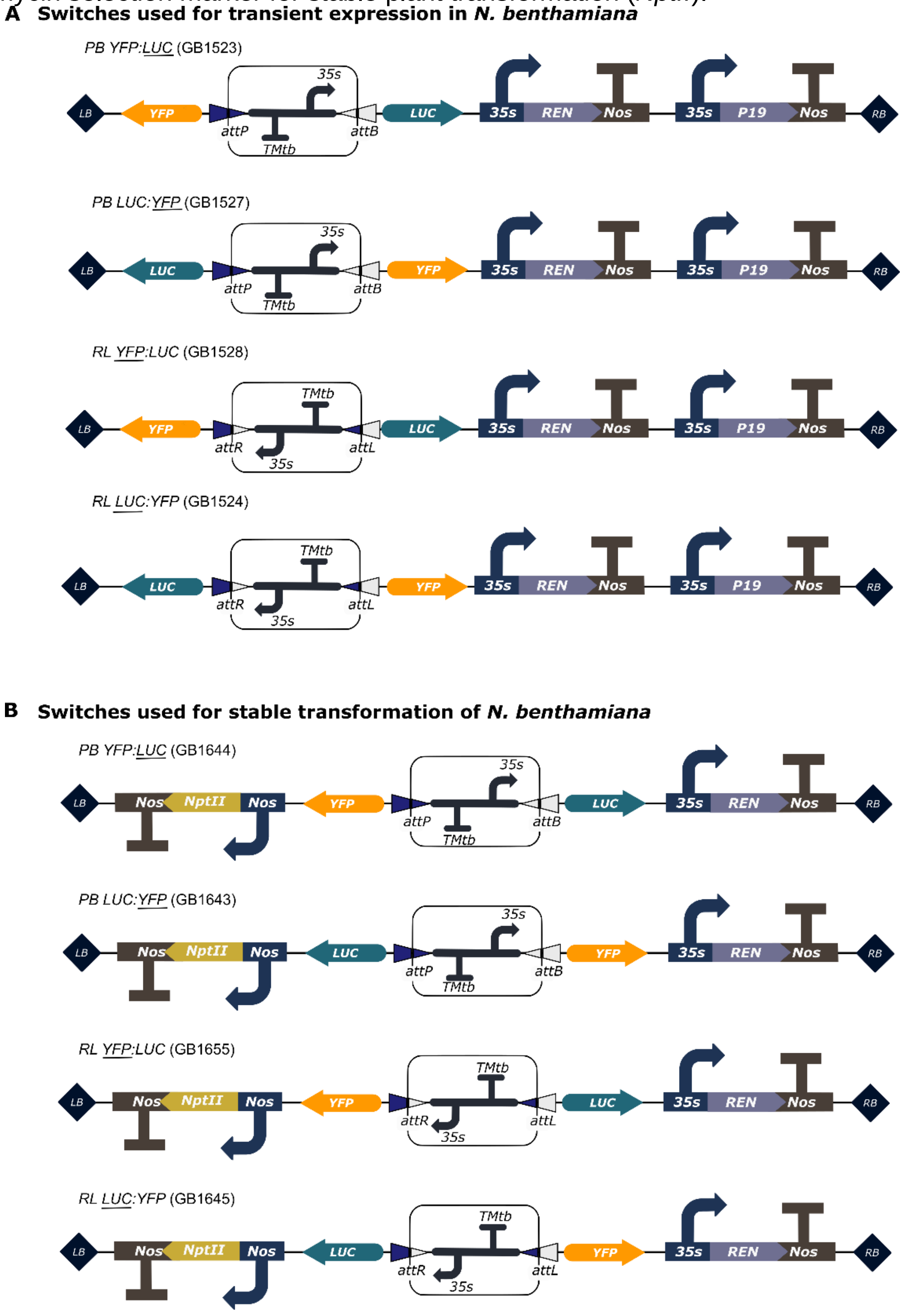

Figure S3: Evaluation of the SET and RESET operations in the genetic switches by transient expression in WT *N. benthamiana* leaves. (A-B) SET and RESET of the *PB LUC:YFP* (GB1523) and *RL LUC:YFP* (GB1524) switches. **(C-D)** SET and RESET of the *PB YFP:LUC* (GB1527) and *RL YFP:LUC* (GB1528) switches. **(A, D)** The recombination processes switch on Fluc reporter expression increasing the Fluc/Rluc ratios (Rluc is expressed as a constitutive internal control). **(B, C)** The recombination reactions switch off Fluc expression resulting in a decrease of the Fluc activity. All the graphs represent the evolution of Fluc/Rluc ratios at intervals of 12 hours post infiltration (hpi) for 96h. Leaves were agroinfiltrated with the Int construct (GB1531) or the Int+RDF construct (GB1508) (blue line) or with an empty vector named as stuffer fragment (SF) used as negative control (C-, grey line). Each point represents the mean of Fluc/Rluc ± SD of three leaves in different plants.

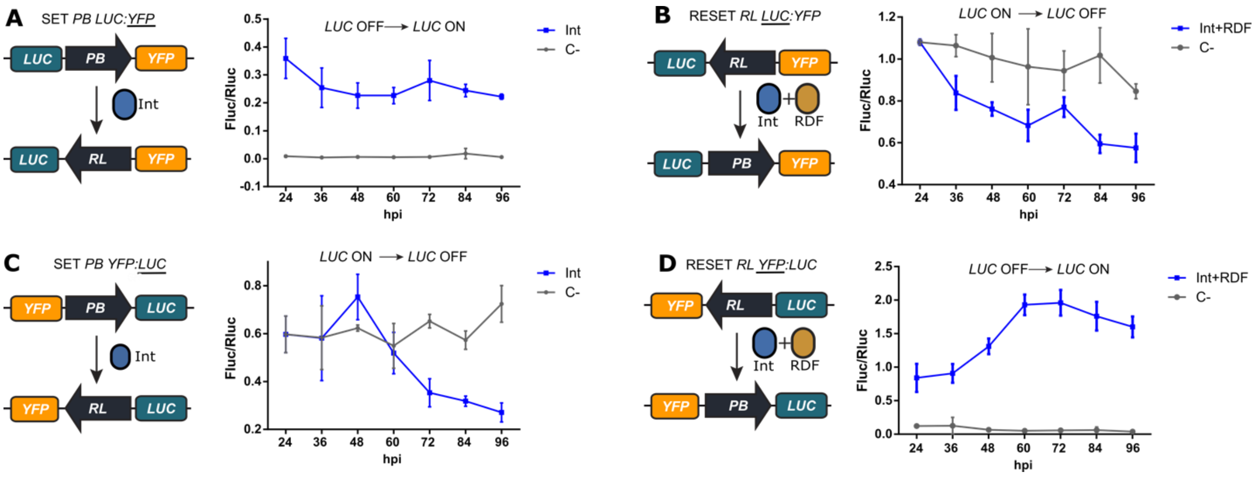

Figure S4: Phenotyping results of the transgenic lines obtained after transformation with the switch constructs. Both the luminescence (Fluc) and the fluorescence intensity (FI) were measured for each line and are indicated in arbitrary units (a.u.). Marked lines (i.e., A2, B5, C4, and D20) were selected for further experiments in the T1 generation. The bars show the mean values ± SD of three replicates.

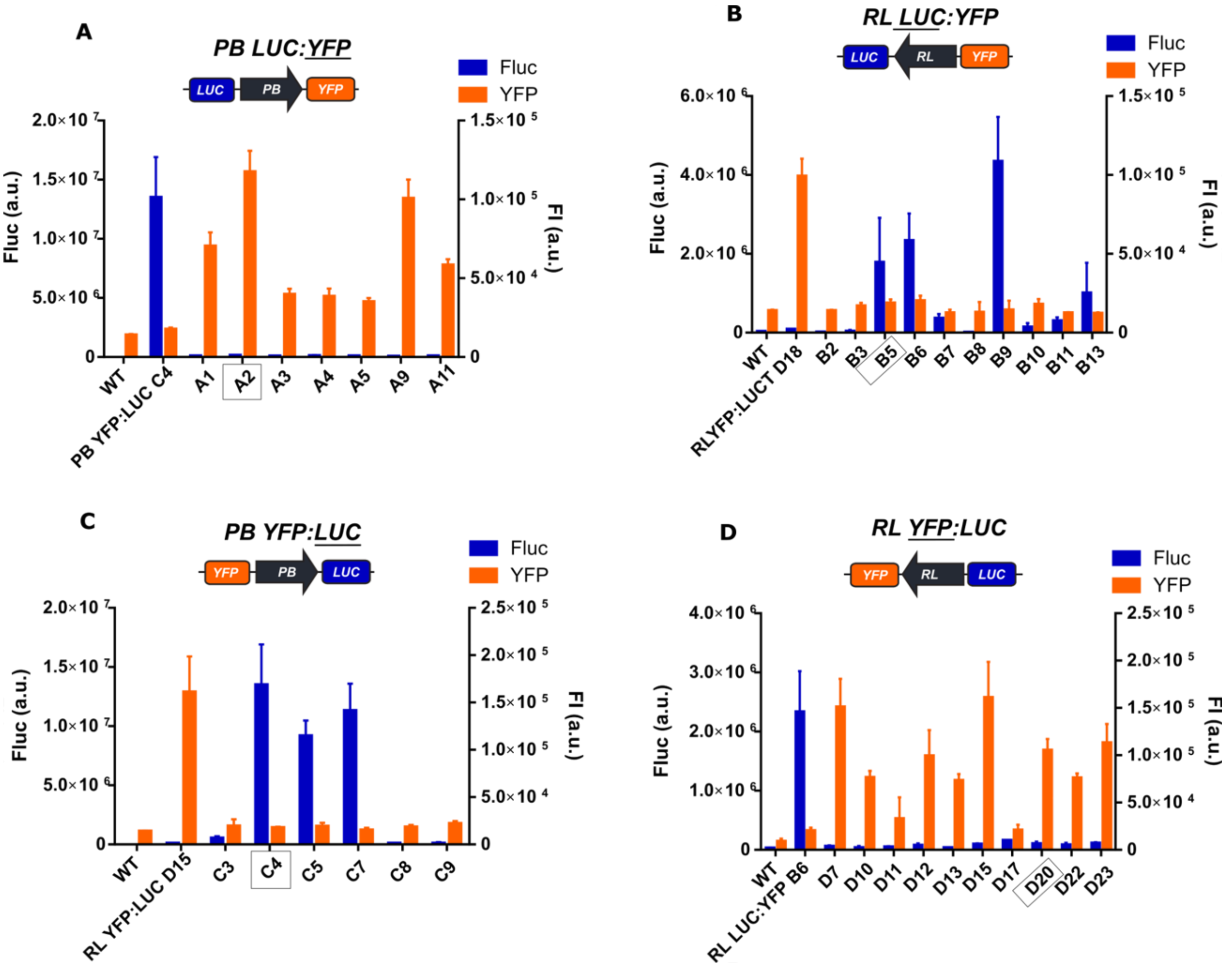

Figure S5: Influence of the optical density (OD) of the *A. tumefaciens* cultures carrying the Int or the Int+RDF on the switching efficiency of stably transformed constructs. **A)** Effect of the OD of Int cultures in the SET process of the *PB LUC:YFP* T1-2 line. **B)** Effect of the OD of Int cultures in the SET process of the *PB YFP:LUC* T1-4 line. **C)** Effect of the ratio of Int:RDF on the reset of the *RL LUC:YFP* T1-5 line. **D)** Effect of the ratio of Int:RDF on the reset of the *RL YFP:LUC* T1-20 line. Different Int:RDF proportions (e.g., 1:1 or 1:2) are achieved by keeping the OD of the Int culture constant at 0.1, and modulating the OD of the RDF cultures as indicated in Table S3. Bars indicate the fold change between the Fluc/Rluc ratios of treated (Int or Int+RDF) and the control samples (P19-infiltrated), expressed as mean ± SD of three agroinfiltrated leaves.

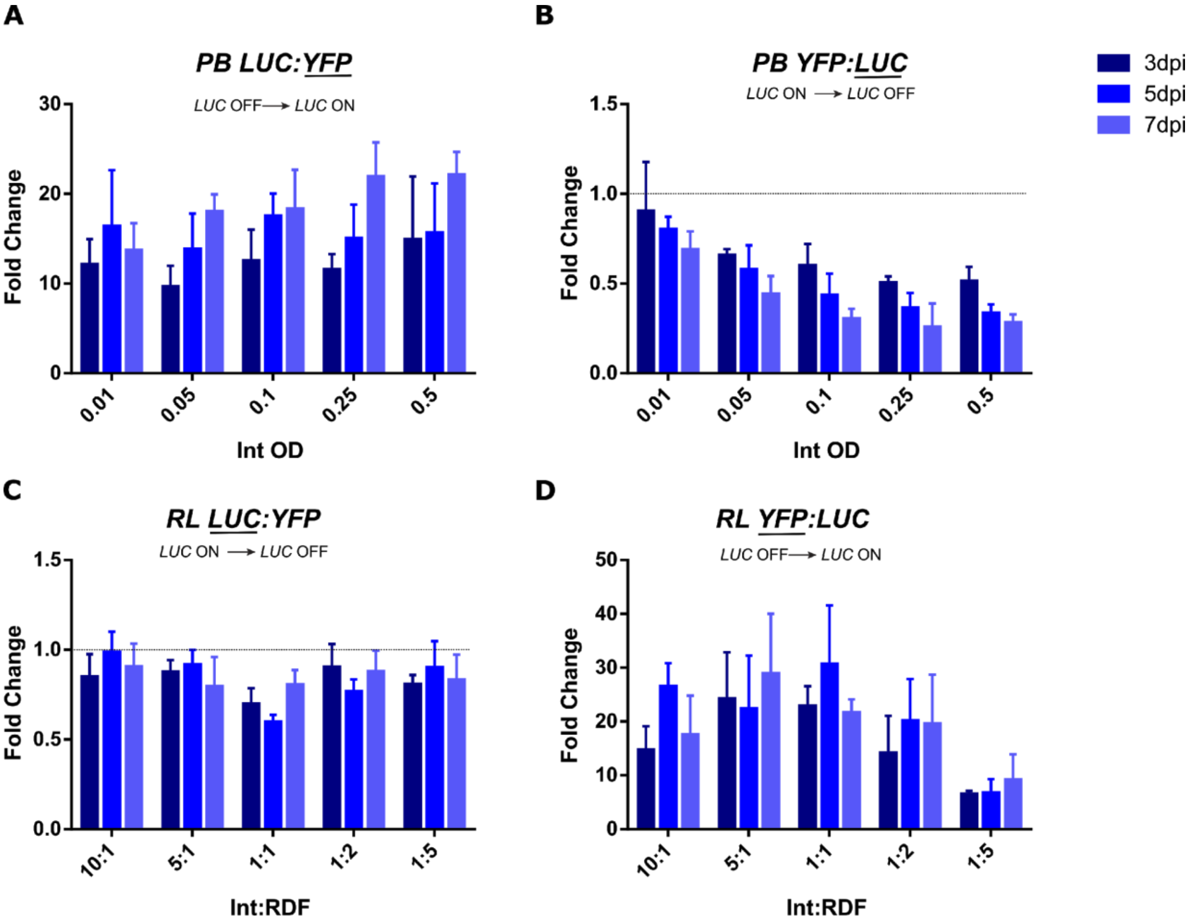

Figure S6: Quantification of the fluorescence intensity (FI) of the confocal images shown in Fig. 4. Bars show the mean FI ± SD of nine images. ND stands for “not detected”.

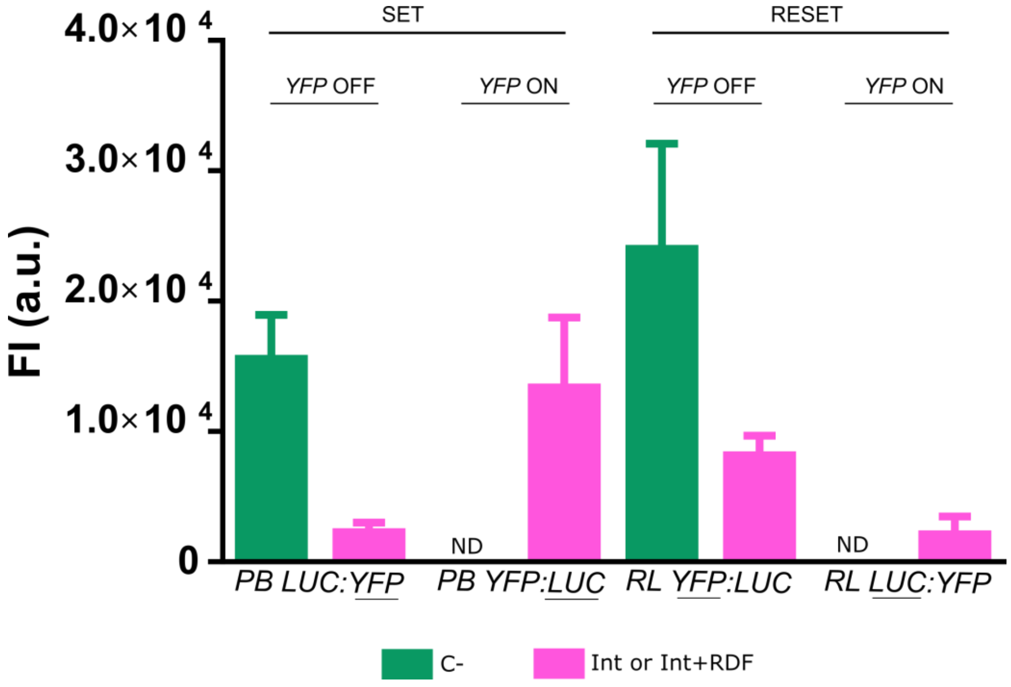

Figure S7: Densitometry of luminescence signal shown in Fig. 7C. **(A)** Quantification of the luminescence signal displayed by estradiol-inducible (EI LUC) and Int-inducible (EI Int) roots incubated for 3 days in estradiol-free (Mock) or estradiol-containing (Estradiol) MS plates. **(B)** Quantification over the same roots 7 days after the estradiol removal. ND means no detected.

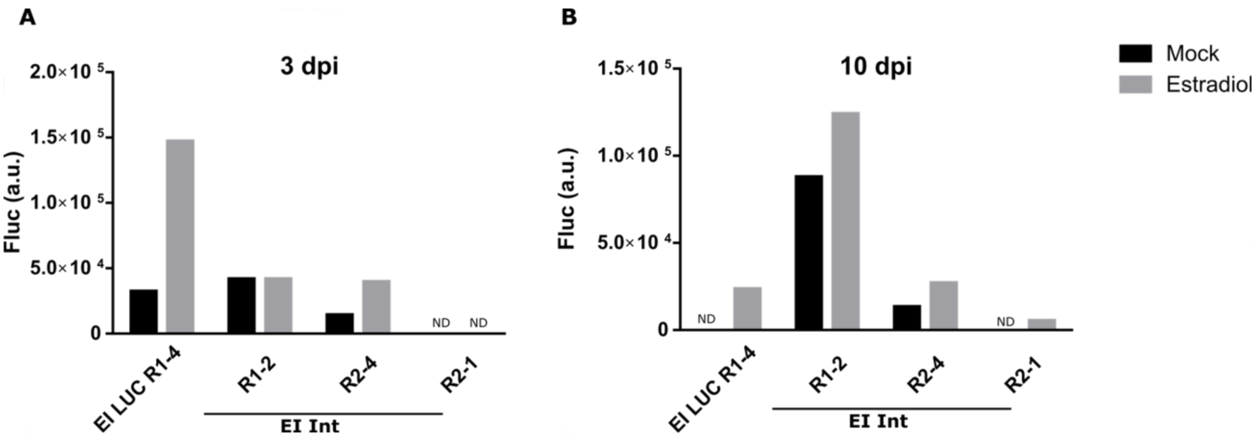

Supplement: gkaa104_Supplemental_Files [file gkaa104_supplemental_files.zip › Supplementary Figures.pdf]
