# Supplementary material for: A memory switch for plant synthetic biology based on the phage ϕC31 integration system
Source: Nucleic Acids Res. 2020 Feb 21;48(6):3379–94. doi: 10.1093/nar/gkaa104 (PMC7102980; doi:10.1093/nar/gkaa104)
Supplement: gkaa104_Supplemental_Files [file gkaa104_supplemental_files.zip › Supplementary Materials and Methods.pdf]

Supplementary materials and methods

This supplementary materials and methods section includes an overview of the assembly method to build a reversible memory switch based on the phage ϕC31 integration system for plant systems. For a detailed description of the GoldenBraid cloning schema and its assembly reactions please see (1-3).

To maximize the exchangeability and reusability of the genetic switch, it is structured in three standard and interchangeable parts, as described in Fig. S1: (i) the coding sequence to the right of the switch encoding *GOI2* (gene of interest 2), (ii) the PB or RL invertible switches, and (iii) the coding sequence to the left of the switch encoding *GOI1* (gene of interest 1). PB and RL states of the switch are already available in the GoldenBraid biorepository (GB1494 and GB1506, Table S1). GoldenBraid users can find multiple Level 0 DNA parts to use them as *GOI1* or *GOI2*, but these can also be constructed *de novo* by the user following the domestication process if the user needs to use new DNA elements. Using these DNA parts, GoldenBraid users can assemble transcriptional units in the Level 1 employing the regulatory sequences (promoters and terminators) available at GoldenBraid or, again, made by themselves. Detailed protocols for Level 0 and Level 1 reactions are described in the references above.

Once a desired transcriptional unit is assembled in Level 1, it can be used as a template for the non-conventional domestication and the subsequent assembly with the PB or RL switch states as depicted below:

1) Level 1 assembly of the transcriptional units encoding *GOI1* and *GOI2* through a BsaI-mediated restriction-ligation reaction in a single tube containing three Level 0 parts (promoter, coding sequence of *GOI*, and terminator) and a pDGB3 alpha destination vector. Below each part, between brackets, the codes of each DNA part, which indicate the overhangs that define each DNA part and that need to be used according to the plant synthetic biology standard if the user is cloning a new DNA part (3,4).

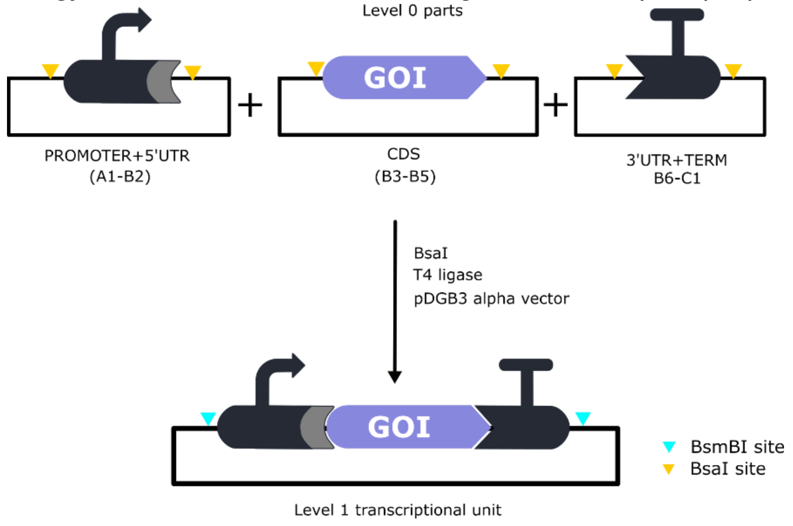

2) Non-conventional domestication of *GOI* involves the amplification of the *GOI* using a previously assembled transcriptional unit (step 1) and a pair of primers containing (i) the BsmBI recognition sequence, (ii) the pUPD2 plasmid overhang (OH), (iii) the overhangs that define *GOI1* and *GOI2* and (iv) a 20-nucleotide priming sequence that anneal with the promoter or the terminator. An existing transcriptional unit comprising the *CaMV35S* promoter and the *TNos* terminator can be domesticated into a *GOI1* or *GOI2* elements by using different sets of primers listed in Table S2: (i) GOI1 - ALF15DIC08 and ALF15DIC10; (ii) GOI2 - ALF15DIC09 and ALF15DIC13. The resulting PCR product is subsequently cloned at Level 0 through a BsmBI-mediated restriction-ligation reaction using pUPD2 as destination plasmid.

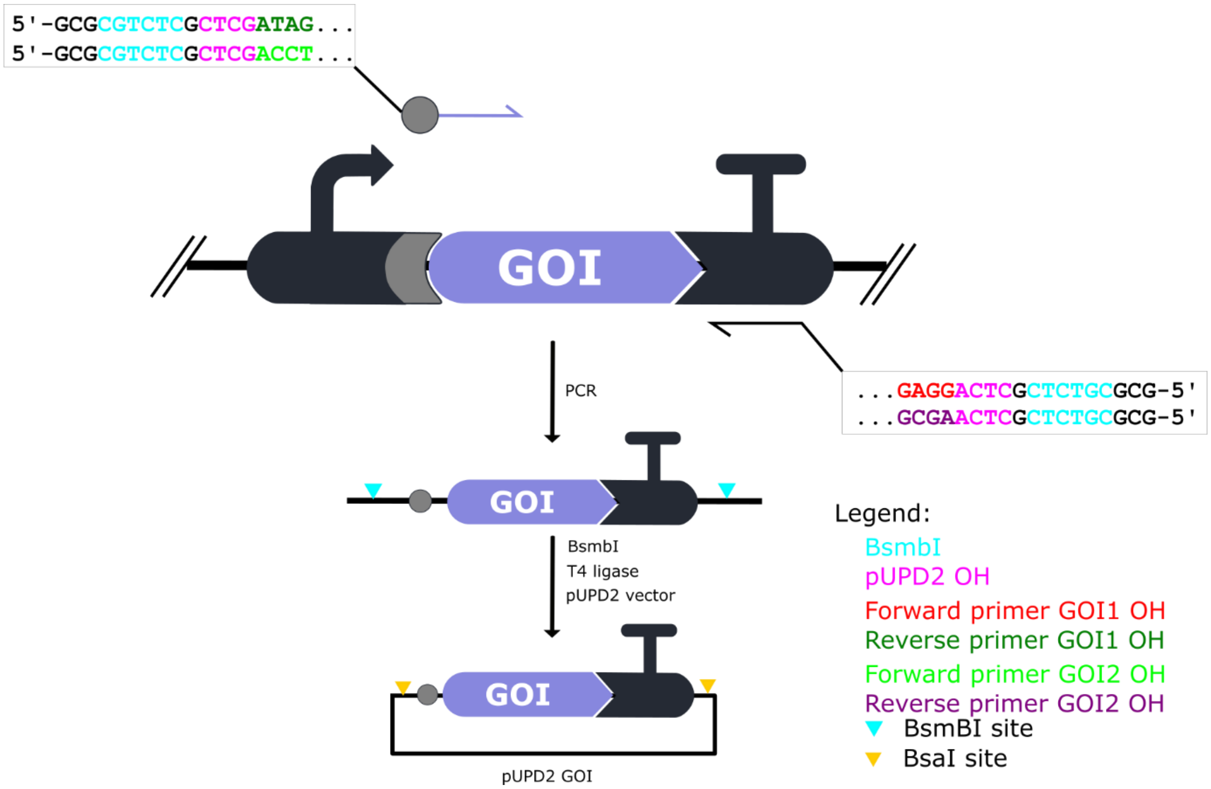

3) Assembly of the reversible switch in Level 1 using the *GOI1* and *GOI2* cloned in the previous step and the PB (GB1494) or RL (GB1506) switch states.

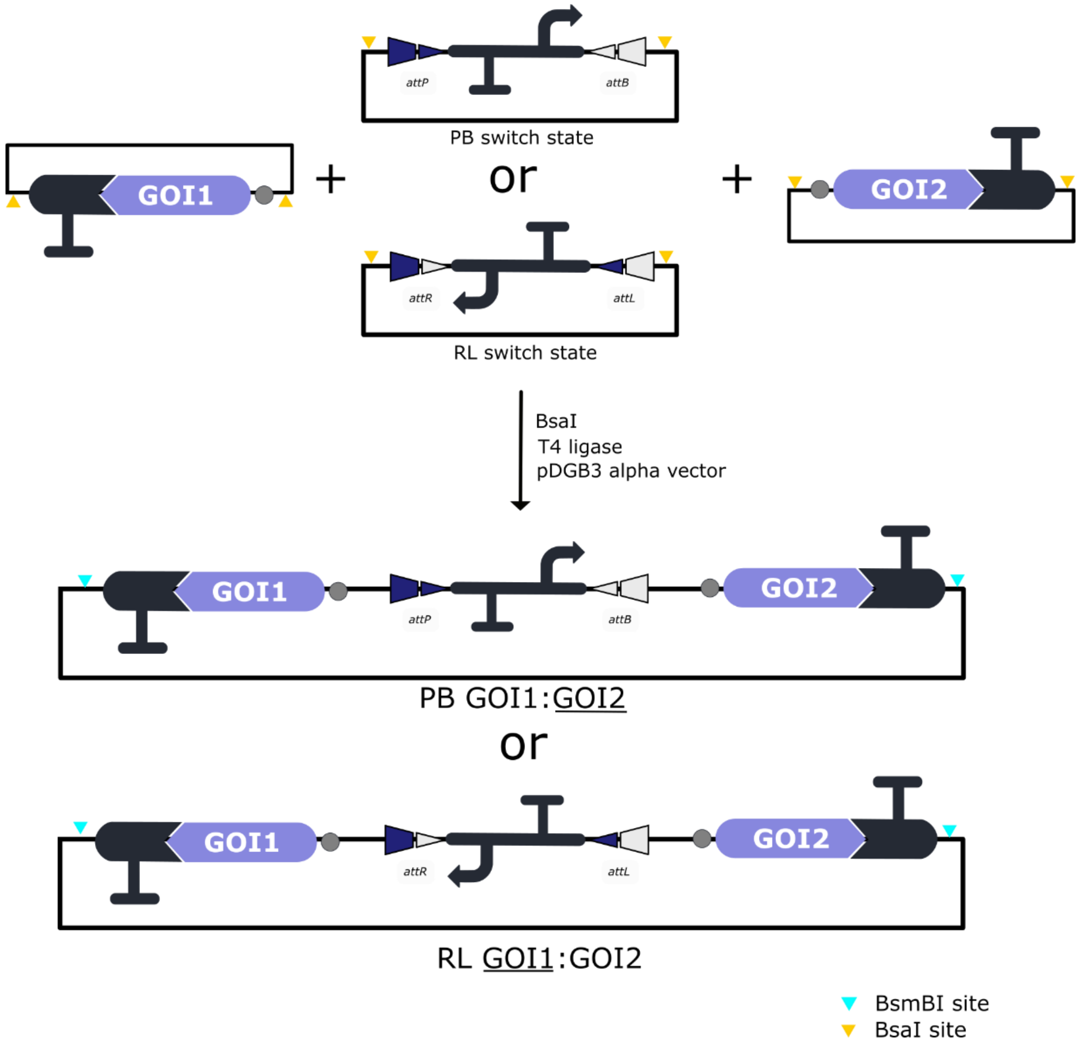

1. Sarrion-Perdigones, A., Vazquez-Vilar, M., Palací, J., Castelijns, B., Forment, J., Ziarsolo, P., Blanca, J., Granell, A. and Orzaez, D. (2013) GoldenBraid 2.0: a comprehensive DNA assembly framework for plant synthetic biology. *Plant Physiol.*, **162**, 1618-1631.
2. Vazquez-Vilar, M., Sarrion-Perdigones, A., Ziarsolo, P., Blanca, J., Granell, A. and Orzaez, D. (2015), *Plant Functional Genomics*. Springer, pp. 399-420.
3. Vazquez-Vilar, M., Quijano-Rubio, A., Fernandez-Del-Carmen, A., Sarrion-Perdigones, A., Ochoa-Fernandez, R., Ziarsolo, P., Blanca, J., Granell, A. and Orzaez, D. (2017) GB3.0: a platform for plant bio-design that connects functional DNA elements with associated biological data. *Nucleic Acids Res.*, **45**, 2196-2209.
4. Patron, N.J., Orzaez, D., Marillonnet, S., Warzecha, H., Matthewman, C., Youles, M., Raitskin, O., Leveau, A., Farré, G., Rogers, C. *et al.* (2015) Standards for plant synthetic biology: a common syntax for exchange of DNA parts. *New Phytol.*, **208**, 13-19.
