# Supplementary material for: A memory switch for plant synthetic biology based on the phage ϕC31 integration system
Source: Nucleic Acids Res. 2020 Feb 21;48(6):3379–94. doi: 10.1093/nar/gkaa104 (PMC7102980; doi:10.1093/nar/gkaa104)
Supplement: gkaa104_Supplemental_Files [file gkaa104_supplemental_files.zip › Supplementary Tables.pdf]

Table S1: Constructs generated in this study. Sequences are accessible at GB cloning website using the GB database ID.

Level 0 GB phytobricks		
**GB database ID**	**Name**	**Category**
1481	Assembly Switch 1/3 Part- T35S:DsRed:5'UTR	Other
1483	Assembly Switch 3/3 Part - 5'UTR:YFP:Tnos	Other
1494	Assembly Switch 2/3 Part - ΦC31 PB (attP:TMtb:P35S:attB)	Other
1496	ΦC31 integrase (Plant Codon Optimized)	B3-B4-B5
1498	RDF (gp3)	B3-B4-B5
1499	Assembly Switch 1/3 Part - TNos:Luciferase:5'UTR	Other
1500	Assembly Switch 3/3 Part - 5'UTR:Luc:Tnos	Other
1506	Assembly Switch 2/3 Part - ΦC31 RL (attR:P35S:TMtb:attL)	Other
1507	Switch 1/3 Tnos:YFP:5'UTR	Other
1561	ΦC31 integrase for C-terminal fusions	B3-B4
2892	Linker-RDF	B5

Level ≥1 GB phytobricks			
**GB database ID**	**Name**	**Category**	**Used in**
108	P35S:P19:T35S	TU	Fig. 2,3, 4,5,6,S4
460	P35S:DsRED:T35s-SF	Module	Intermediary
1129	P35S:ER:LexADBD/Gal4AD:T35S	TU	Intermediary
1130	OpLexA:mini35S:Luciferase:T35S	TU	Intermediary
1131	P35S:ER:LexABD/Gal4AD:T35S-OpLexA:mini35S:Luciferase:T35S	Module	Intermediary
1495	Switch ΦC31 PB (DsRed:YFP)	Other	Fig. S2, S3
1497	P35S:ϕC31:T35S	TU	Intermediary

1508	P35S:RDF:T35S	TU	Fig. 2,3,4,5
1510	Switch ϕC31 RL (DsRed:YFP)	Other	Fig. S2, S3
1513	Switch ϕC31 PB (Luc:YFP)	Other	Fig. S2
1514	Switch ϕC31 RL (Luc:YFP)	Other	Fig. S2
1517	Switch ϕC31 PB (YFP:Luc)	Other	Fig. S2
1518	Switch ϕC31 RL (YFP:Luc)	Other	Fig. S2
1523	Switch PB ϕC31 Luc:YFP-P35S:Renilla:Tnos-P35S:p19:Tnos	Module	Fig. S2, S3
1524	Switch RL ϕC31 Luc:YFP-P35S:Renilla:Tnos-P35S:p19:Tnos	Module	Fig. S2, S3
1527	Switch PB ϕC31 YFP:Luc-P35S:Renilla:Tnos-P35S:p19:Tnos	Module	Fig. S2, S3
1528	Switch RL ϕC31 YFP:Luc-P35S:Renilla:Tnos-P35S:p19:Tnos	Module	Fig. S2, S3
1529	OplexA:mini35S:ϕC31:Tnos	TU	Intermediary
1531	Pnos:ϕC31:Tnos	TU	Fig. 2,3,4,5,6
1532	P35S:ER:lexABD/Gal4AD:T35S - OplexA:mini35S:ϕC31:Tnos	Module	Intermediary
1601	Pnos:ER:LexABD:GAL4AD:Tnos	TU	Intermediary
1643	Tnos:nptII:Pnos-PB LUC:YFP-P35S:Rluc:T35S	Module	Fig. 4
1644	Tnos:nptII:Pnos-PB YFP:LUC-P35S:Rluc:T35S	Module	Fig. 4
1645	Tnos:nptII:Pnos-RL LUC:YFP-P35S:Rluc:T35S	Module	Fig. 4
1655	Tnos:nptII:Pnos-RL YFP:LUC-P35S:Rluc:T35S	Module	Fig. 4
1677	Pnos:ER:lexABD:GAL4AD:T35s-OplexA:mini35S:ϕC31:Tnos	Module	Intermediary
2060	Pnos:ϕC31:Tnos - 35S:RDF:T35S	Module	Intermediary
2313	Pnos:ER:LexABD:GAL4AD:T35S-OpLexA:mini35S:ΦC31:Tnos-35S:DsRED:T35S – SF	Module	Fig. 7
2388	35S:ER:LexABD:GAL4AD:T35S-OpLexA:mini35S:LUC:Tnos-35S:DsRED:T35S – SF	Module	Fig. 7
2893	P35S:ϕC31-RDF:T35S	TU	Fig. 5

Table S2: oligonucleotides used in this study.

Name	Sequence (5'-3')
JO18SEP01 RL YFPLUC F	CAGAGCAGAGATCATGGTGTTAG
JO18SEP02 RL YFPLUCR	GCATACGACGATTCTGTGATTTG
JO18SEP05 BP YFPLUC F	TTGTGGCTGTTGTAGTTGTACTC
JO18SEP06 BP YFPLUCR	ATCATGGTGTTAGCCTTCTATGG
JO18SEP03 PhiC31 F	GTTGAATTAGACTGTGGACCGAT
JO18SEP04 PhiC31 R	ATCTTGTGCATCGTCTTCATCAT
ALF15EN04	GCGCGTCTCGACGAAAATATAGTTGAAACAGA
ALF15EN05	GCGCGTCTCGTCGTACTAGAGCCAAGCTGATCTC
ALF15NOV03	GCGCGTCTCGCTCGCTATAGTAGTGCCCCAACTGGGGTAACCTTTGAGTTCTCTCAGTTGGGGGCGTA GTCGCAAAAACTATATGCTCT
ALF15NOV04	GCGCGTCTCGCTCAAGGTCGGTGCGGGTGCCAGGGCGTGCCCTTGGGCTCCCCGGGCGCGTACTCC ACTAGTAAATTGTAATGTTGTTTGTTG
ALF15DIC06	GCGCGTCTCGCTCGCTATAGTAGTGCCCCAACTGGG
ALF15DIC07	GCGCGTCTCGCTCAAGGTCGGTGCGGGTGCCA
ALF15DIC08	GCGCGTCTCGCTCGATAGAAACAACATTACAATTTACTATTCTAGTCGA
ALF15DIC09	GCGCGTCTCGCTCGACCTAAACAACATTACAATTTACTATTCTAGTCGA
ALF15DIC10	GCGCGTCTCGCTCAGGAGCGAGTCGGTCCCATT
ALF15DIC11	GCGCGTCTCGCTCAGGAGAGGTCACTGGATTTTGGTTTTAGG
ALF15DIC12	GCGCGTCTCGCTCAAGCGAGGTCACTGGATTTTGGTTTTAGG
ALF15DIC13	GCGCGTCTCGCTCAAGCGCGAGTCGGTCCCATT
MV1 F1	AACTGGGGTAACCTTTGGGCTCC
MV2 R1	CCACTATCCTTCGCAAGACCCTTCC
MV3 F2	CGGAAAGACGATGACGGAAA
MV4 R2	CGGTACTTCGTCCACAAACA

Table S3: Table S3 shows the relative volumes of the different agroinfiltration cultures and their respective concentrations (measured as OD600) that were employed to formulate the agroinfiltration mixes used in all switching experiments, including optimization experiments shown in Fig. S5 (columns 1 and 2).

Optimization of ΦC31		Optimization of ΦC31 + RDF		PB switch kinetics		RL switch kinetics		RL switch kinetics with ΦC31-RDF fusion	
**Culture**	**OD**	**Culture**	**OD**	**Culture**	**OD**	**Culture**	**OD**	**Culture**	**OD**
1 volume ΦC31	0.01	1 volume ΦC31	0.1	1 volume ΦC31	0.1	1 volume ΦC31	0.1	1 vol. ΦC31-RDF	0.1
	0.05	1 volume RDF	0.01	NA	NA	1 volume RDF	0.1		
	0.1		0.05	NA	NA				
	0.25		0.1	NA	NA				
	0.5		0.2	NA	NA				
			0.5	NA	NA				
1 volume P19	0.1	1 volume P19	0.1	2 volumes P19	0.1	1 volume P19	0.1	2 vol.. P19	0.1
